# The importance of comprehensive geriatric assessment in predicting the outcome of patients with proximal humerus fractures

**DOI:** 10.1007/s40520-026-03357-9

**Published:** 2026-03-05

**Authors:** Jan-Philipp Happe, J. Christoph Katthagen, Karen Fischhuber, Ursula Marschall, Andreas Faldum, Michael J. Raschke, Jeanette Koeppe, Josef Stolberg-Stolberg

**Affiliations:** 1 Research group Mathematical Surgery, University Hospital Muenster, University of Muenster, Muenster, Germany; 2 Department of Trauma, Hand and Reconstructive Surgery, University Hospital Muenster, Albert-Schweitzer-Campus 1, Building W1, 48149 Muenster, Germany; 3 Institute of Biostatistics and Clinical Research, University of Muenster, Muenster, Germany; 4 BARMER Institute for Health System Research, Wuppertal, Germany

**Keywords:** Proximal Humeral Fracture, Geriatric, Multimorbidity, surgical treatment

## Abstract

**Background:**

Proximal humeral fractures (PHF) are common in geriatric patients. Existing frailty scores insufficiently capture the multidimensional vulnerability influencing long-term outcomes.

**Aims:**

This study investigates if geriatric-typical characteristic complexes (GTMK), incorporating a broader range of geriatric domains, can improve prediction of long-term outcomes following PHF.

**Methods:**

In this retrospective cohort study, patients aged ≥ 65 years with PHF (ICD-10 S42.2) were identified from 2011 to 2022 using nationwide inpatient and outpatient claims data from the German BARMER health insurance database. Treatment type, GTMK, and multiple comorbidities of the patients were collected. Long-term outcomes including overall survival, major adverse events, thromboembolic events, surgical complications, and minor outpatient complications were analyzed using multivariable Cox regression and competing risk models.

**Results:**

A total of 91,189 patients with PHF were analyzed, of whom 42.9% underwent surgical treatment. The cohort was predominantly female (84.2%) with a median age of 78 years. An increasing number of GTMK was strongly associated with higher long-term risks across all outcomes. Strong individual predictors included malnutrition for mortality (HR 1.43; 95% CI 1.36–1.51), decubital ulcers for major adverse events (HR 1.31; 95% CI 1.27–1.36), and malnutrition for thromboembolic events (HR 1.39; 95% CI 1.32–1.46).

**Discussion:**

Geriatric-typical characteristics captured vulnerabilities not reflected by traditional frailty measures and substantially influenced post-fracture prognosis, highlighting their clinical relevance in risk stratification.

**Conclusion:**

The GTMKs *malnutrition*,* decubital ulcers*,* cognitive deficits*,* and fluid/electrolyte disorders* consistently emerged as the strongest predictors for long term adverse outcomes across multiple endpoints.

**Supplementary Information:**

The online version contains supplementary material available at 10.1007/s40520-026-03357-9.

## Introduction

Proximal humeral fractures (PHF) are among the most common injuries in geriatric patients and typically result from low-energy falls associated with osteoporosis, sarcopenia, and impaired balance [1, 2]. Due to the continuous growth of the aging population, the incidence of PHF is steadily increasing, leading to significant clinical and socioeconomic challenges [1]. Recovery of function after PHF is essential for maintaining independence, preserving quality of life, and reducing long-term care dependency in older adults [3]. Consequently, reliable prediction of long-term outcomes is crucial to support individualized treatment decisions.

Research on frailty in orthopaedic surgery is highly heterogeneous, with a wide variety of frailty assessment tools used across different subspecialties and outcome measures [4–10]. Although no universally accepted definition or gold-standard frailty instrument exists, several frailty indices have been shown to be associated with adverse postoperative outcomes [11] . One of the most used tools is the Modified 5-Item Frailty Index (mFI-5), particularly in surgical populations [12–14]. However, the mFI-5 is limited to five comorbidity-based variables, functional dependence, diabetes mellitus, chronic obstructive pulmonary disease, congestive heart failure, and hypertension and therefore reflects only a narrow aspect of the multidimensional frailty concept. In addition, most frailty scores, including the mFI-5, have primarily been validated for short-term perioperative outcomes and provide limited information on long-term functional recovery [15–18].

Geriatric-typical characteristic complexes (GTMK) are ICD-based diagnostic codes representing conditions commonly observed in geriatric patients; the presence of two or more GTMK defines geriatric-typical multimorbidity (GTMM), by which a patient is classified as geriatric [19]. GTMKs encompass a broader range of geriatric-relevant factors, including cognitive impairment, sarcopenia, malnutrition, polypharmacy, psychosocial vulnerability, functional decline, and reduced physiological reserve. These dimensions are highly relevant in geriatric care but are frequently underrepresented or omitted in traditional frailty assessment models [20–22]. By capturing multiple domains of vulnerability, GTMK may allow for a more precise and clinically meaningful risk stratification, particularly regarding long-term outcomes following PHF [3].

The aim of the present study is to evaluate the predictive value of GTMK for long-term outcomes in geriatric patients with proximal humeral fractures.

## Methods

This study utilized retrospective in-patient and out-patient claims data provided by BARMER, which is one of Germany’s largest statutory health insurance providers, covering approximately 8 million insured individuals nationwide. All inpatient hospitalizations coded for PHF, identified using the ICD-10 code S42.2, were included. Outpatient consultations with the same diagnostic code were also considered. The study period included all cases recorded between January 2011 and December 2022. All eligible records identified during this time frame were extracted from the database for further analysis.

### Patient cohort

**Fig. 1:**
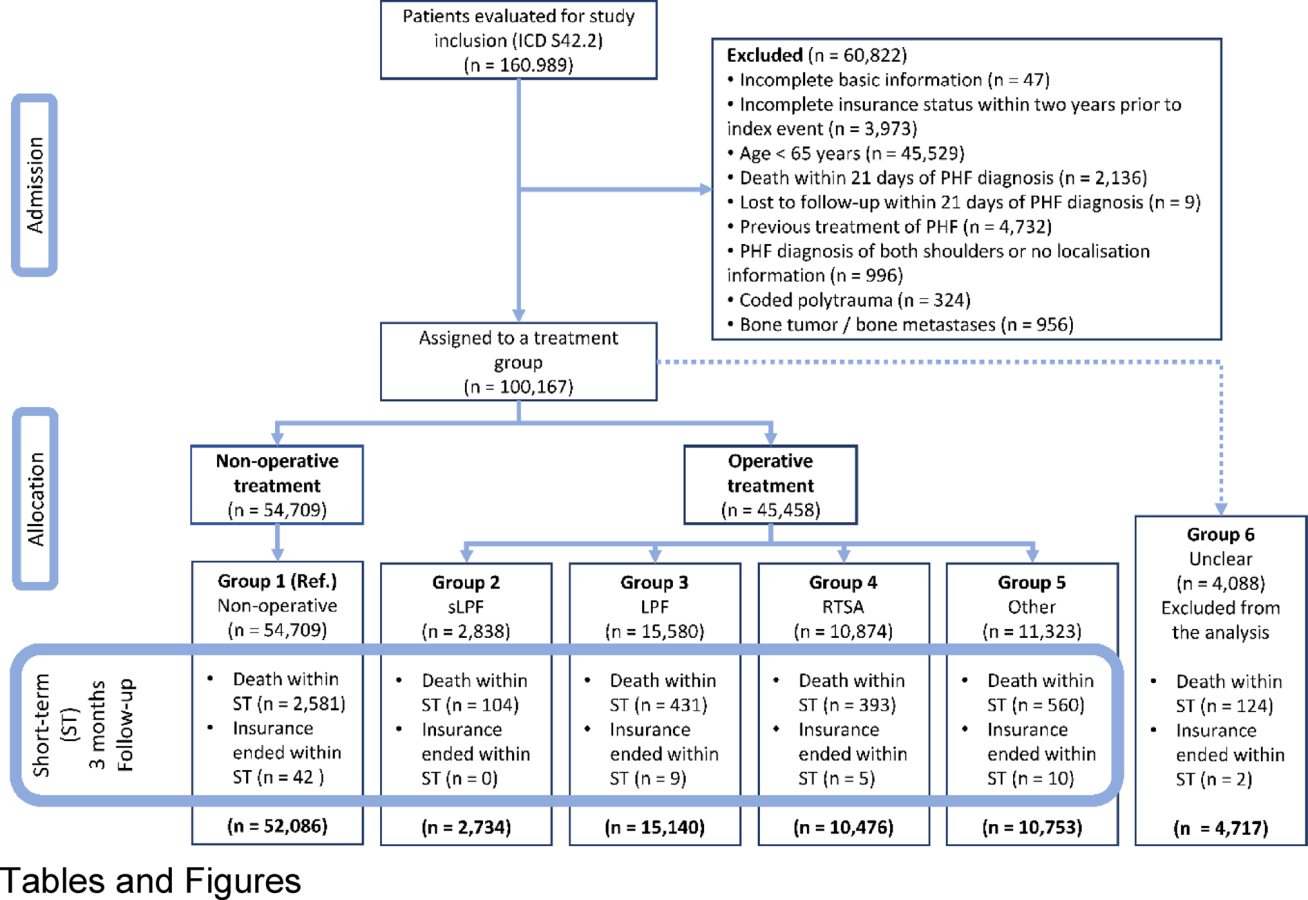
CONSORT flow-chart. Admission of suitable patients and their allocation to one of the treatment groups ‘non-operative’, ‘operative’ with a locked plate fixation for simple (sLPF) and multi-fragment fracture (LPF), reverse total shoulder arthroplasty (RTSA), as well as ‘other’ or ‘not clearly assignable’ (unclear) operative treatment. For this long-term analysis, patients with a follow-up duration shorter than 3 months (short-term) were excluded. Unclear operative treatment is also excluded from the analysis

The study included all patients who were 65 years of age or older at the time of their first recorded PHF diagnosis. To be eligible for inclusion, patients had to have at least one inpatient or outpatient encounter coded with ICD-10 S42.2 during the study period. The study’s flowchart with a complete list of exclusion criteria is presented in Fig. 1.

Surgical interventions were identified based on specific OPS procedure codes. Patients who underwent reverse total shoulder arthroplasty (RTSA) were identified using OPS code 5-824.21. Those who received locked plate fixation for multi-fragmentary fractures were identified using OPS codes 5-794.k1 or 5-794.21 (LPF). Patients who underwent locked plate fixation for simple fractures were identified using OPS codes 5-793.k1 or 5-793.31 (sLPF). Additional surgical procedures were categorized separately, and a detailed list of these procedures is provided in Supplementary Table S1.

Patients were subsequently assigned to one of five treatment groups based on the surgical procedure received, or lack thereof, within the first 21 days following the initial PHF diagnosis. For further Information refer to Table 1.

**Table 1:** Patient characteristics of the cohort at the time of PHF diagnosis. Patients were assigned to a non-operative or operative treatment group based on treatment within the first 21 days after PHF diagnosis. For this long-term analysis, patients with a follow-up duration shorter than 3 months (short-term) were excluded (see Fig. 1). sLPF - locked plate fixation for simple fracture, LPF - locked plated fixation for multi-fragment fracture, PHF - proximal humeral fracture, RTSA -reverse total shoulder arthroplasty

Frequency – *N* (%)	Total cohort (Group 1–5)	Non-operative (Group 1)	Operative (Group 2–5)	sLPF (Group 2)	LPF (Group 3)	RTSA (Group 4)	Others (Group 5)	Unclear (Group 6)
	91,189 (100.0%)	52,086 (57.1%)	39,103 (42.9%)	2,734 (3.0%)	15,140 (16.6%)	10,476 (11.5%)	10,753 (11.8%)	4,717 (5.2%)
Age in years – Median (Q1, Q3)	78 (72, 84)	79 (72, 85)	78 (72, 83)	77 (71, 83)	77 (71, 82)	80 (74, 84)	78 (72, 84)	76 (71, 82)
Over 70 years – N (%)	76,154 (83.5%)	43,487 (83.5%)	32,667 (83.5%)	2,199 (80.4%)	12,066 (79.7%)	9,426 (90.0%)	8,976 (83.5%)	3,703 (78.5%)
Geriatric – N (%)	63,340 (69.5%)	36,513 (70.1%)	26,827 (68.6%)	1,812 (66.3%)	9,760 (64.5%)	7,871 (75.1%)	7,384 (68.7%)	3,012 (63.9%)
Female – N (%)	76,792 (84.2%)	43,275 (83.1%)	33,517 (85.7%)	2,356 (86.2%)	13,075 (86.4%)	9,136 (87.2%)	8,950 (83.2%)	4,054 (85.9%)
**Comorbidities**								
Atrial fibrillation and atrial flutter – N (%)	16,813 (18.4%)	9,975 (19.2%)	6,838 (17.5%)	417 (15.3%)	2,335 (15.4%)	2,062 (19.7%)	2,024 (18.8%)	701 (14.9%)
Alcohol abuse – N (%)	4,373 (4.8%)	2,229 (4.3%)	2,144 (5.5%)	137 (5.0%)	699 (4.6%)	609 (5.8%)	699 (6.5%)	224 (4.7%)
Atherosclerosis – N (%)	14,857 (16.3%)	8,613 (16.5%)	6,244 (16.0%)	455 (16.6%)	2,272 (15.0%)	1,804 (17.2%)	1,713 (15.9%)	733 (15.5%)
Bisphosphonate – N (%)	5,996 (6.6%)	3,317 (6.4%)	2,679 (6.9%)	184 (6.7%)	1,015 (6.7%)	730 (7.0%)	750 (7.0%)	315 (6.7%)
Cancer – N (%)	24,883 (27.3%)	14,370 (27.6%)	10,513 (26.9%)	779 (28.5%)	3,952 (26.1%)	2,959 (28.2%)	2,823 (26.3%)	1,310 (27.8%)
Congestive heart failure – N (%)	21,145 (23.2%)	12,313 (23.6%)	8,832 (22.6%)	556 (20.3%)	2,977 (19.7%)	2,623 (25.0%)	2,676 (24.9%)	851 (18.0%)
Chronic polyarthritis – N (%)	5,895 (6.5%)	3,400 (6.5%)	2,495 (6.4%)	166 (6.1%)	949 (6.3%)	698 (6.7%)	682 (6.3%)	271 (5.7%)
Chronic kidney disease – N (%)	21,674 (23.8%)	12,397 (23.8%)	9,277 (23.7%)	584 (21.4%)	3,103 (20.5%)	2,901 (27.7%)	2,689 (25%)	952 (20.2%)
Dementia – N (%)	11,348 (12.4%)	7,192 (13.8%)	4,156 (10.6%)	289 (10.6%)	1,403 (9.3%)	1,057 (10.1%)	1,407 (13.1%)	375 (7.9%)
Diabetes – N (%)	27,434 (30.1%)	15,633 (30.0%)	11,801 (30.2%)	717 (26.2%)	4,316 (28.5%)	3,491 (33.3%)	3,277 (30.5%)	1,227 (26.0%)
Frozen shoulder – N (%)	3,968 (4.4%)	2,428 (4.7%)	1,540 (3.9%)	89 (3.3%)	633 (4.2%)	419 (4.0%)	399 (3.7%)	187 (4%)
Any anticoagulant – N (%)	28,356 (31.1%)	17,128 (32.9%)	11,228 (28.7%)	749 (27.4%)	4,014 (26.5%)	3,292 (31.4%)	3,173 (29.5%)	1,218 (25.8%)
Hypertonus – N (%)	75,038 (82.3%)	42,636 (81.9%)	32,402 (82.9%)	2,193 (80.2%)	12,209 (80.6%)	9,043 (86.3%)	8,957 (83.3%)	3,713 (78.7%)
Coronary heart disease – N (%)	23,011 (25.2%)	13,638 (26.2%)	9,373 (24.0%)	606 (22.2%)	3,403 (22.5%)	2,617 (25.0%)	2,747 (25.5%)	1,012 (21.5%)
Nicotine abuse – N (%)	5,869 (6.4%)	3,175 (6.1%)	2,694 (6.9%)	216 (7.9%)	989 (6.5%)	744 (7.1%)	745 (6.9%)	376 (8.0%)
Obesity – N (%)	18,667 (20.5%)	10,247 (19.7%)	8,420 (21.5%)	476 (17.4%)	3,104 (20.5%)	2,682 (25.6%)	2,158 (20.1%)	999 (21.2%)
Omarthrosis – N (%)	2,700 (3.0%)	1,761 (3.4%)	939 (2.4%)	49 (1.8%)	322 (2.1%)	317 (3.0%)	251 (2.3%)	120 (2.5%)
Any osteoporosis medication – N (%)	11,024 (12.1%)	6,329 (12.2%)	4,695 (12.0%)	323 (11.8%)	1,725 (11.4%)	1,347 (12.9%)	1,300 (12.1%)	564 (12.0%)
Osteoporosis – N (%)	31,565 (34.6%)	17,688 (34.0%)	13,877 (35.5%)	913 (33.4%)	5,132 (33.9%)	4,078 (38.9%)	3,754 (34.9%)	1,563 (33.1%)
Parkinson – N (%)	3,545 (3.9%)	2,090 (4.0%)	1,455 (3.7%)	94 (3.4%)	486 (3.2%)	398 (3.8%)	477 (4.4%)	132 (2.8%)
Previous Stroke – N (%)	24,364 (26.7%)	14,534 (27.9%)	9,830 (25.1%)	686 (25.1%)	3,581 (23.7%)	2,754 (26.3%)	2,809 (26.1%)	1,136 (24.1%)
Rotator cuff rupture – N (%)	2,434 (2.7%)	1,160 (2.2%)	1,274 (3.3%)	60 (2.2%)	428 (2.8%)	414 (4.0%)	372 (3.5%)	132 (2.8%)
Vitamin D or Calcium – N (%)	6,415 (7.0%)	3,788 (7.3%)	2,627 (6.7%)	179 (6.5%)	934 (6.2%)	819 (7.8%)	695 (6.5%)	349 (7.4%)
**Geriatrics-typical characteristic complexes (GTMK)**								
Immobility – N (%)	2,903 (3.2%)	1,784 (3.4%)	1,119 (2.9%)	82 (3.0%)	349 (2.3%)	346 (3.3%)	342 (3.2%)	123 (2.6%)
Falling tendency – N (%)	32,888 (36.1%)	19,331 (37.1%)	13,557 (34.7%)	925 (33.8%)	4,766 (31.5%)	4,178 (39.9%)	3,688 (34.3%)	1,554 (32.9%)
Cognitive deficits – N (%)	12,496 (13.7%)	7,925 (15.2%)	4,571 (11.7%)	322 (11.8%)	1,533 (10.1%)	1,181 (11.3%)	1,535 (14.3%)	434 (9.2%)
Incontinence – N (%)	23,093 (25.3%)	13,980 (26.8%)	9,113 (23.3%)	647 (23.7%)	3,214 (21.2%)	2,586 (24.7%)	2,666 (24.8%)	945 (20.0%)
Decubital ulcers – N (%)	7,312 (8.0%)	4,484 (8.6%)	2,828 (7.2%)	185 (6.8%)	927 (6.1%)	767 (7.3%)	949 (8.8%)	280 (5.9%)
Malnutrition and malnourishment – N (%)	2,195 (2.4%)	1,319 (2.5%)	876 (2.2%)	69 (2.5%)	304 (2.0%)	198 (1.9%)	305 (2.8%)	79 (1.7%)
Fluids and electrolytes disorders – N (%)	23,277 (25.5%)	13,782 (26.5%)	9,495 (24.3%)	638 (23.3%)	3,315 (21.9%)	2,684 (25.6%)	2,858 (26.6%)	995 (21.1%)
Depression and anxiety disorders – N (%)	31,101 (34.1%)	17,919 (34.4%)	13,182 (33.7%)	927 (33.9%)	4,995 (33.0%)	3,628 (34.6%)	3,632 (33.8%)	1,593 (33.8%)
Pain – N (%)	36,355 (39.9%)	21,266 (40.8%)	15,089 (38.6%)	1,035 (37.9%)	5,660 (37.4%)	4,366 (41.7%)	4,028 (37.5%)	1,781 (37.8%)
Sensory disorders – N (%)	25,560 (28.0%)	14,791 (28.4%)	10,769 (27.5%)	697 (25.5%)	4,034 (26.6%)	3,152 (30.1%)	2,886 (26.8%)	1,246 (26.4%)
Frailty – N (%)	10,791 (11.8%)	6,466 (12.4%)	4,325 (11.1%)	250 (9.1%)	1,416 (9.4%)	1,583 (15.1%)	1,076 (10%)	466 (9.9%)
Severe visual and hearing impairment – N (%)	47,440 (52%)	27,338 (52.5%)	20,102 (51.4%)	1,376 (50.3%)	7,749 (51.2%)	5,503 (52.5%)	5,474 (50.9%)	2,438 (51.7%)
Medication problems – N (%)	654 (0.7%)	380 (0.7%)	274 (0.7%)	18 (0.7%)	103 (0.7%)	64 (0.6%)	89 (0.8%)	31 (0.7%)
High risk of complications – N (%)	63,094 (69.2%)	35,971 (69.1%)	27,123 (69.4%)	1,906 (69.7%)	10,327 (68.2%)	7,376 (70.4%)	7,514 (69.9%)	3,216 (68.2%)
Delayed convalescence– N (%)	380 (0.4%)	208 (0.4%)	172 (0.4%)	6 (0.2%)	53 (0.4%)	56 (0.5%)	57 (0.5%)	13 (0.3%)
**Number of geriatric-typical characteristic complexes (GTMK) present at PHF diagnosis**								
Mean (sd)	3.50 (2.21)	3.59 (2.24)	3.39 (2.16)	3.32 (2.14)	3.22 (2.10)	3.60 (2.18)	3.45 (2.21)	3.22 (2.08)
Median (1. Quantile, 3. Quantile)	3 (2, 5)	3 (2, 5)	3 (2, 5)	3 (2, 5)	3 (2, 5)	3 (2, 5)	3 (2, 5)	3 (2, 5)
No GTMK – N (%)	6,225 (6.8%)	3,406 (6.5%)	2,819 (7.2%)	209 (7.6%)	1,207 (8.0%)	646 (6.2%)	757 (7.0%)	375 (7.9%)
1 GTMK – N (%)	11,692 (12.8%)	6,474 (12.4%)	5,218 (13.3%)	357 (13.1%)	2,218 (14.6%)	1,211 (11.6%)	1,432 (13.3%)	669 (14.2%)
2 GTMK – N (%)	15,285 (16.8%)	8,445 (16.2%)	6,840 (17.5%)	526 (19.2%)	2,762 (18.2%)	1,702 (16.2%)	1,850 (17.2%)	891 (18.9%)
3 GTMK – N (%)	16,189 (17.8%)	9,059 (17.4%)	7,130 (18.2%)	484 (17.7%)	2,853 (18.8%)	1,867 (17.8%)	1,926 (17.9%)	839 (17.8%)
4 GTMK – N (%)	13,965 (15.3%)	7,966 (15.3%)	5,999 (15.3%)	399 (14.6%)	2,260 (14.9%)	1,698 (16.2%)	1,642 (15.3%)	746 (15.8%)
5 GTMK – N (%)	10,933 (12%)	6,406 (12.3%)	4,527 (11.6%)	324 (11.9%)	1,637 (10.8%)	1,364 (13%)	1,202 (11.2%)	521 (11.0%)
6 GTMK – N (%)	7,592 (8.3%)	4,553 (8.7%)	3,039 (7.8%)	216 (7.9%)	1,049 (6.9%)	899 (8.6%)	875 (8.1%)	334 (7.1%)
7 GTMK – N (%)	4,723 (5.2%)	2,866 (5.5%)	1,857 (4.7%)	108 (4.0%)	639 (4.2%)	571 (5.5%)	539 (5.0%)	194 (4.1%)
8 GTMK – N (%)	2,660 (2.9%)	1,683 (3.2%)	977 (2.5%)	69 (2.5%)	300 (2.0%)	309 (2.9%)	299 (2.8%)	87 (1.8%)
≤ 9 GTMK – N (%)	1,925 (2.1%)	1,228 (2.4%)	697 (1.8%)	42 (1.5%)	215 (1.4%)	209 (2.0%)	231 (2.1%)	61 (1.3%)

### Confounder model

Comorbidity profiles, prior medication use, and previous medical procedures were assessed using diagnostic and procedural codes documented during the five years prior to the initial PHF diagnosis. Variables related to GTMM were collected using inpatient and outpatient data recorded during the two years prior to the index fracture diagnosis.

Different time windows were applied for specific conditions. Acute pain conditions were evaluated within the three months preceding the PHF diagnosis, while prior fracture history was assessed within a 26-week period before the index event. A complete list of all diagnostic and procedural codes used to define the study variables is provided in Supplementary Table S1.

### Primary and secondary endpoints

Primary and secondary endpoints were assessed in patients who remained event-free during the initial 3-month (short-term) period and were followed until the end of follow-up (December 2022). The primary outcomes were overall survival (OS) and major adverse events (MAE). Secondary outcomes included thromboembolic events (TE), injury- or surgery-related complications (SC), and minor outpatient complications (MOC).

### Statistical methods

All analyses were performed using anonymized routine claims data, which ensured near-complete data capture for all clinical and procedural variables. Since the dataset as well as the definition of all variables was based entirely on coded diagnoses (ICD-10) and procedures (OPS), no missing information occurred, aside from occasional inconsistencies in basic demographic data (such as sex, date of birth, or insurance coverage periods). In cases where no relevant diagnostic or procedural code was recorded for a particular variable, this was interpreted as absence of the condition or procedure and coded accordingly.

For this long-term outcome analysis, patients were followed beyond the early postoperative phase to capture extended recovery trajectories. Treatment assignment was based on interventions performed within 21 days following the initial diagnosis of PHF, allowing sufficient time for definitive surgical management while maintaining consistency across the cohort. Patients who died within this initial 21-day period were excluded from analysis; however, any medical events occurring during this period were incorporated into the multivariable models as part of the baseline risk profile. To specifically evaluate long-term outcomes, only patients who did not experience any of the predefined primary endpoints (OS, MAE, TE, SC, or MOC) within the first 3 months after PHF diagnosis were included in this analysis.

Survival time was calculated starting from 3 months and 21 days after the index PHF diagnosis. Time-to-event analyses for OS and MAE were performed using Kaplan-Meier estimates for unadjusted event rates, and Cox proportional hazards models for multivariable analysis. Hazard ratios (HR) with corresponding 95% confidence intervals (CI) were calculated and visualized using forest plots. For endpoints with competing risks-specifically SC and MOC, where death precludes the occurrence of the event - cumulative incidence functions were estimated using the Aalen-Johansen method, and subdistributional hazard models according to Fine and Gray were applied for multivariable competing risk analysis.

The multivariable models were adjusted for treatment group, age, sex, year of PHF diagnosis, healthcare sector (inpatient vs. outpatient), comorbidity burden, medication history, events occurring during the initial 21-day period, and the presence of GTMK. The GTMK were included either as individual variables or as cumulative counts of the number of present complexes at the time of PHF diagnosis. To explore whether the impact of GTMK differed between operative and non-operative treatment strategies, interaction terms between GTMK and surgical status were introduced into the models, with corresponding interaction p-values (p_int) reported.

All statistical analyses were considered exploratory and intended to generate hypotheses rather than confirm pre-specified hypotheses. Therefore, p-values < 0.05 were regarded as indicative of potential associations without adjustment for multiple testing. All data processing, statistical modeling, and visualizations were performed using R (version 4.3.1, R Core Team, 2023, Vienna) and SAS Enterprise Guide (version 8.3 Update 2; SAS Institute Inc., Cary, NC, USA).

## Results

### Patient demographics

Between January 2011 and December 2022, 91,189 patients with PHF, complete treatment classification within 21 days of diagnosis and a follow-up of at least 3 months were identified. Surgical management was performed in 42.9% of cases, consisting of LPF (38.7%), RTSA (26.8%), sLPF (7.0%), and other surgical procedures (27.5%). The overall cohort was predominantly female (84.2%) with a median age of 78 years (IQR 72–84). 69.5% fulfilled geriatric criteria which were defined as age ≥ 65 years in combination with the presence of at least two coded GTMKs. The proportion of geriatric patients was comparable between surgically treated (68.6%) and non-operatively managed patients (70.1%).

The most frequently observed GTMK were severe visual and hearing impairment (52.0%), high risk of complications (69.2%), depression and anxiety disorders (34.1%), and falling tendency (36.1%). GTMK prevalence was largely balanced across treatment groups. Incontinence (26.8% vs. 23.3%) and fluid/electrolyte disorders (26.5% vs. 24.3%) were slightly more frequent in the non-operative group. Further details are reported in Table 1.

### Influence of the number of GTMK on primary outcomes

**Fig. 2:**
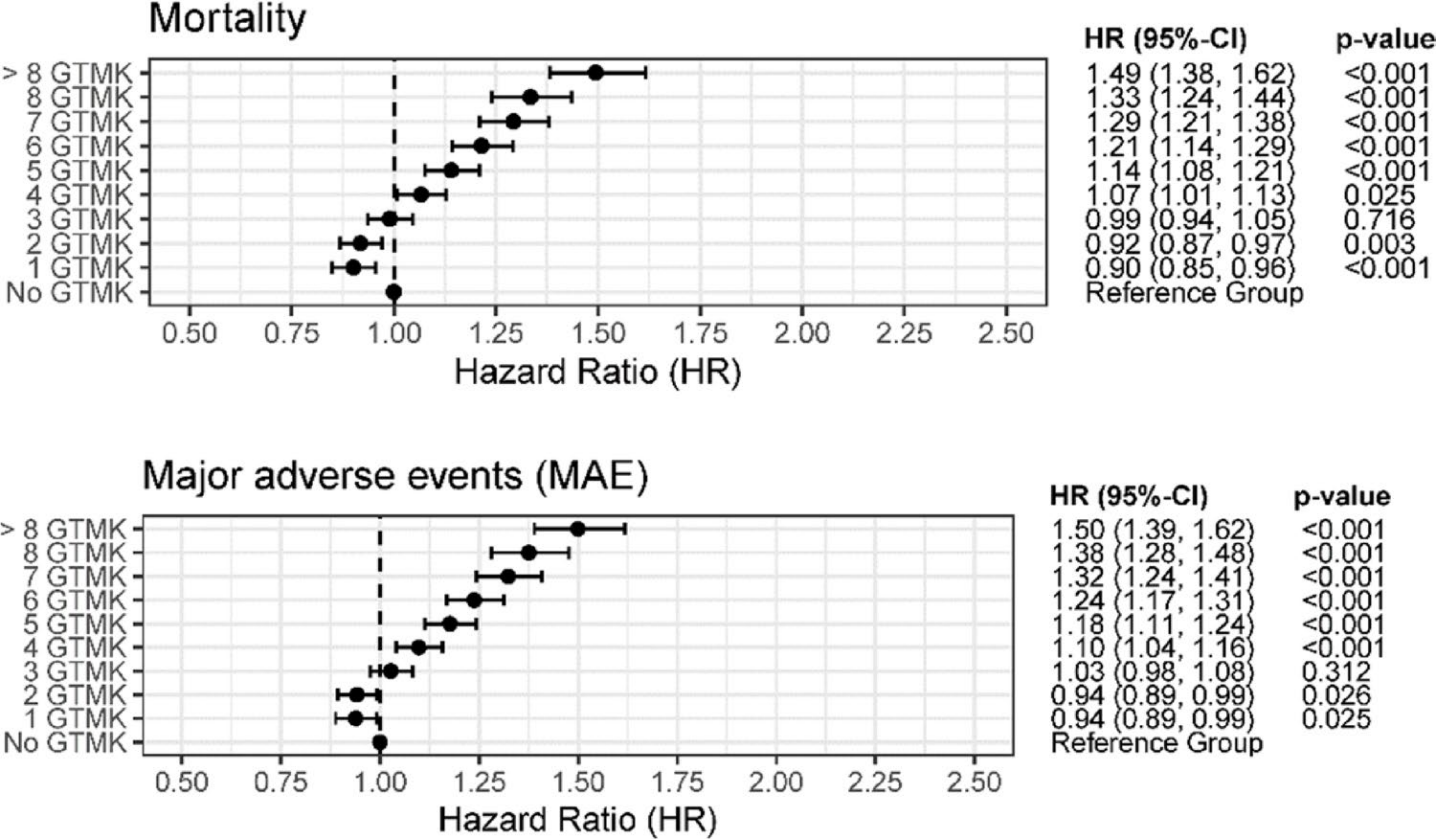
Influence of the number of GTMK present at the time of PHF on the risk of experiencing a primary event, among patients who did not experience any event within the first 3 months. Hazard ratios (HR) were adjusted for patients’ risk profiles using multivariable Cox regression, making these HR estimates independent of the treatment group. Complete regression results are provided in Table S3 to Table S7

Overall, the distribution of the number of GTMKs was similar across treatment groups. Most patients presented with three GTMKs at diagnosis with a consistent median of 3 (IQR 2–5) across surgical subgroups. The mortality risk increased progressively from 4 GTMK onwards, becoming statistically noticeable from 4 GTMK (HR 1.07; 95% CI 1.01–1.13; *p* = 0.025). Interestingly, patients with 1 GTMK showed a notably lower mortality risk compared to the reference group without GTMK (HR 0.90; 95% CI 0.85–0.96; *p* < 0.001). Patients with > 8 GTMK had a higher mortality risk compared to patients without GTMKs (HR 1.49; 95% CI 1.38–1.62; *p* < 0.001). The association between GTMK and the occurrence of MAE showed similar trends compared to mortality. A statistically noticeable risk increase for MAE was observed to start from 4 GTMK onward (HR 1.10; 95% CI 1.04–1.16; *p* < 0.001). Furthermore, patients with 1 or 2 GTMK had a noticeable reduced risk (HR 0.94; 95% CI 0.89–0.99; *p* = 0.025), while patients having > 8 GTMK showed a higher risk for the long-term occurrence of MAE (HR 1.50; 95% CI 1.39–1.62; *p* < 0.001). These findings are summarized in Fig. 2.

### Influence of the Number of GTMK on secondary outcomes

The risk for the occurrence of TE showed similar trends; a noticeable risk-increase became evident at 4 GTMK (HR 1.11; 95% CI 1.05–1.17; *p* < 0.001). Patients with > 8 GTMK had a higher risk of thromboembolic events (HR 1.46; 95% CI 1.36–1.58; *p* < 0.001).

The risk for the occurrence of SC became statistically noticeable from 3 GTMK onwards (HR 1.21; 95% CI 1.06–1.39; *p* = 0.005). The highest risk increase was seen with 7 GTMK (HR 1.43; 95% CI 1.18–1.73; *p* < 0.001), however no noticeable increase was observed for > 8 GTMK (HR 1.31; 95% CI 0.98–1.77; *p* = 0.073) whilst 8 GTMK (HR 1.35; 95% CI 1.06–1.72; *p* = 0.016) reached statistical noticeability.

The risk for MOC increased sharply with the number of GTMK. Even low numbers of GTMK were associated with elevated risk; already at 1 GTMK the risk was increased by 32% (HR 1.32; 95% CI 1.22–1.43; *p* < 0.001). Patients with > 8 GTMK had more than double the risk (HR 2.07; 95% CI 1.76–2.42; *p* < 0.001). Further details are represented in Table S2.

### Influence of individual GTMKs on primary outcomes

To further elucidate the contribution of specific frailty domains to long-term outcomes, individual GTMKs were analyzed for their associations with mortality, MAE, and TE, stratified by surgical and non-surgical treatment groups and adjusted for patient risk profile.

**Fig. 3:**
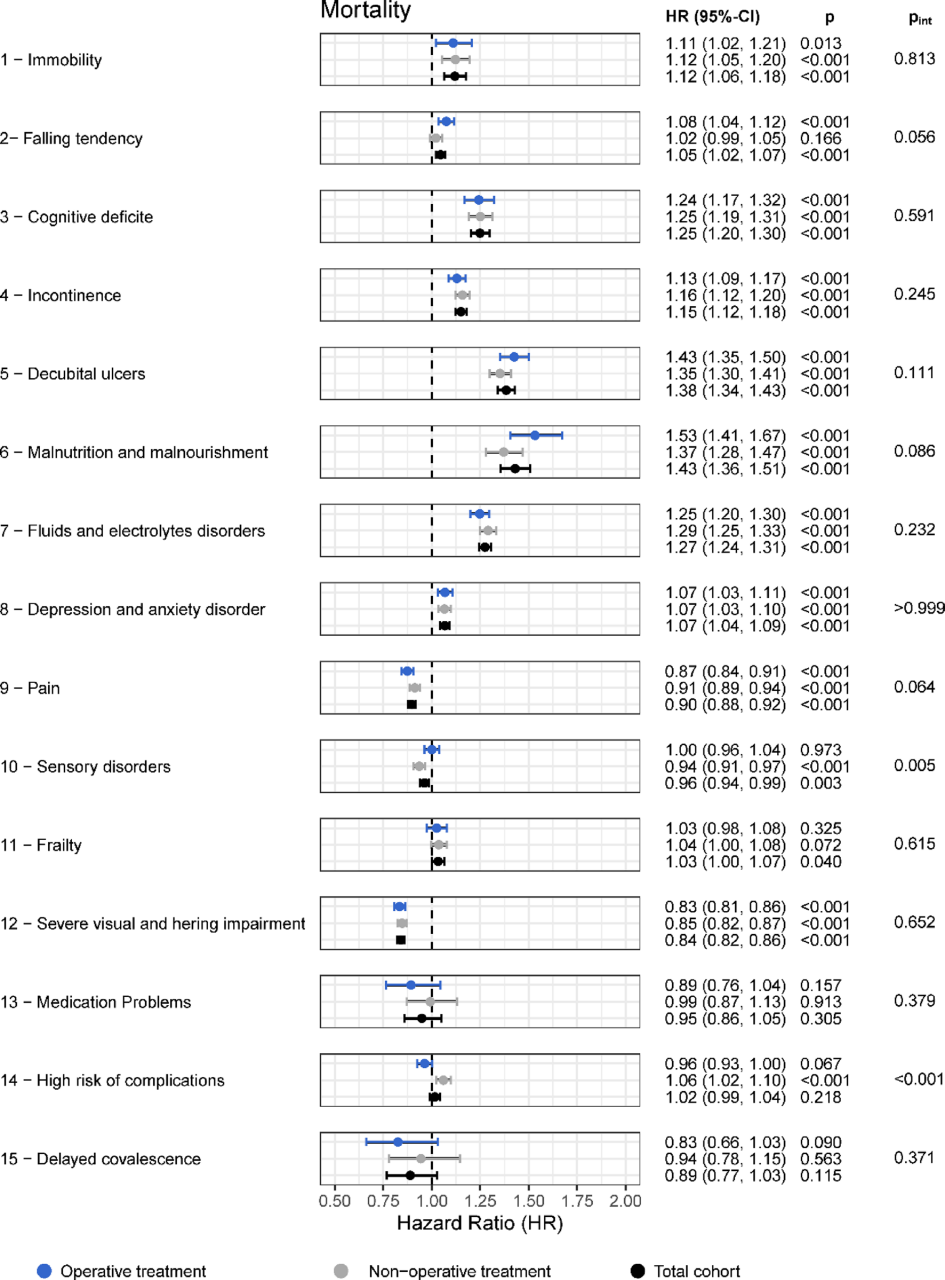
Influence of individual GTMK on Mortality, adjusted for patient risk profile. The analysis was performed separately for non-operative patients (light gray), operative patients (blue), and the overall cohort (black), including only patients who remained event-free after 3 months. Interaction p-values (p_int) were calculated using a Cox regression model with interaction terms between each GTMK and the binary surgery variable, measuring differences in GTMK effects between non-operative and operative subgroups. Complete regression results are provided in Table S8

For mortality, several GTMK domains consistently emerged as strong independent predictors across both treatment modalities. Malnutrition and malnourishment demonstrated the highest risk increase, with an elevated mortality risk in surgically treated patients (HR 1.53; 95% CI 1.41–1.67; *p* < 0.001) and in the overall cohort (HR 1.43; 95% CI 1.36–1.51; *p* < 0.001). Decubital ulcers similarly conferred substantial risk (operative HR 1.43; 95% CI 1.35–1.50; *p* < 0.001; total cohort HR 1.38; 95% CI 1.34–1.43; *p* < 0.001), followed by cognitive deficits, fluid and electrolyte disorders, incontinence, immobility, and depression/anxiety disorders. While numerically higher hazard ratios were often observed among surgically treated patients, interaction testing revealed no statistically significant treatment modifications for most domains, indicating comparable mortality risks across treatment groups. These findings are reported Fig. 3.

For MAE, a similar pattern of dominant risk contributors emerged. Malnutrition (operative HR 1.41; 95% CI 1.30–1.54; *p* < 0.001; total HR 1.38; 95% CI 1.31–1.45; *p* < 0.001), decubital ulcers, cognitive deficits, and electrolyte disorders were again among the strongest predictors. Notably, significant interaction effects were observed for decubital ulcers (p_int = 0.043) and sensory disorders (p_int < 0.001), suggesting that these domains may have differential impact depending on whether surgical intervention was performed. This indicates potential increased vulnerability to MAE in frail surgical candidates presenting with cognitive or metabolic derangements.

### Influence of individual GTMKs on secondary outcomes

For TE, malnutrition (operative HR 1.47; 95% CI 1.35–1.60; *p* < 0.001; total HR 1.39; 95% CI 1.32–1.46; *p* < 0.001) and decubital ulcers (operative HR 1.39; 95% CI 1.32–1.46; *p* < 0.001) again represented the most potent risk factors across both treatment groups. Falling tendencies and sensory disorders significant interaction effects (p_int = 0.038 and p_int = 0.040, respectively), again pointing toward possible treatment-dependent susceptibility in these domains.

Conversely, certain GTMK domains, including sensory disorders and severe visual/hearing impairment, were paradoxically associated with lower risk across multiple endpoints and treatment groups. These findings likely reflect complex confounding factors such as differential treatment allocation, reverse causality, or limitations inherent to administrative coding data. Full results for all domains and interaction testing are detailed in Tables S8–S12.

### Differences in GTMK among surgical vs. non-surgical treated patients

To evaluate the differential impact of GTMKs on long-term outcomes in surgically versus non-surgically treated patients, subgroup analyses were performed for each characteristic.

The number of GTMKs demonstrated a strong dose-dependent association with all evaluated 1-year outcomes. Across all endpoints, increasing GTMK burden corresponded to progressively higher event rates. For mortality, patients treated non-operatively consistently exhibited higher 1-year mortality rates compared to surgically managed patients, particularly among those with 4 or more GTMKs. Similar trends were observed for MAE, TE, SC, and MOC, with incremental risk increases observed for each additional GTMK present at the time of PHF diagnosis. Notably, MOC showed an early and steep increase in risk, even with low GTMK counts, while for mortality and MAE, risk elevations became most pronounced at higher GTMK levels (≥ 4). Across treatment groups, RTSA and LPF were associated with generally lower 1-year event rates for TE, MAE and mortality compared to non-operative management, though this difference narrowed with increasing frailty burden. At the highest GTMK levels (> 8), all treatment groups exhibited high event rates, suggesting a saturation effect in patients with extreme frailty burden. For reference refer to Figure S1.

**Fig. 4:**
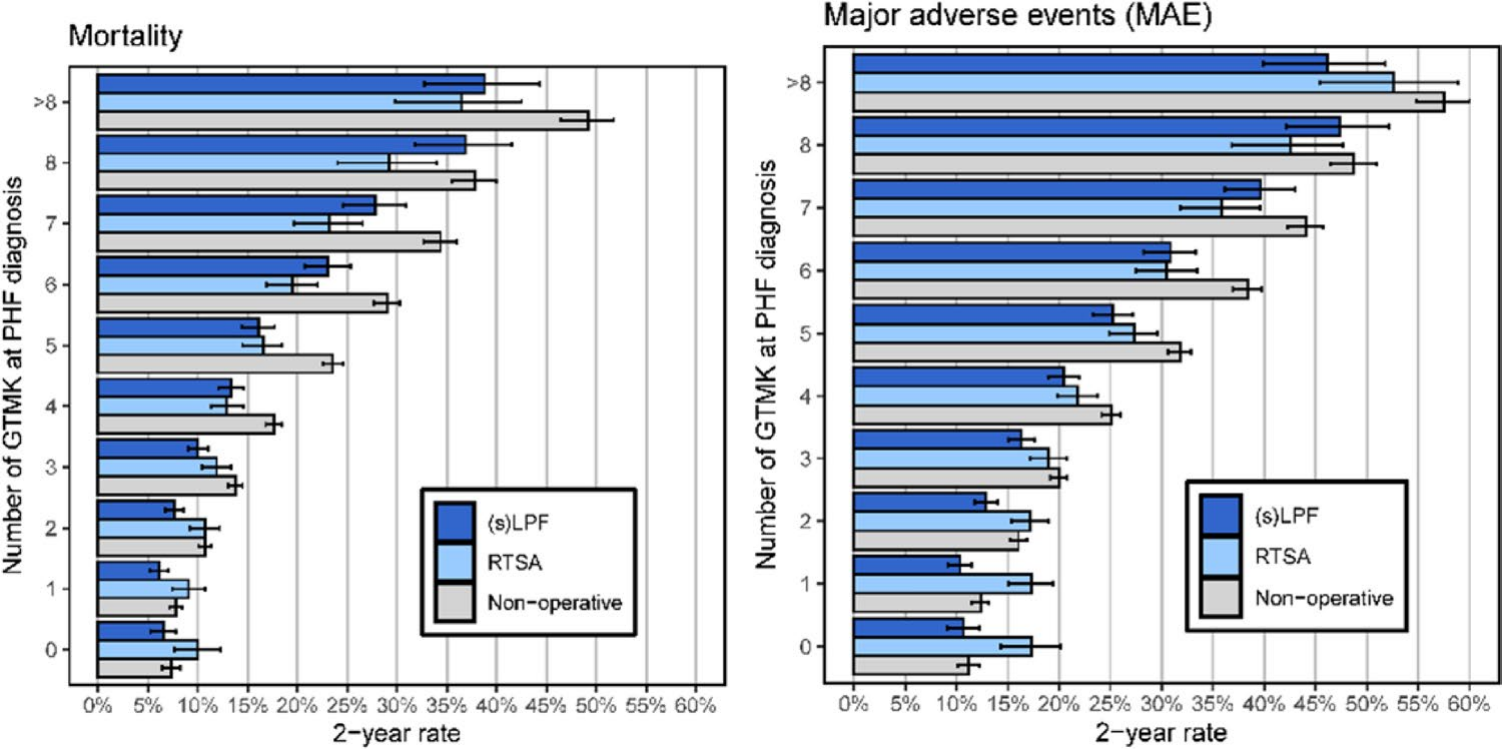
2-year rates with 95% confidence interval determined using Kaplan-Meier estimates of the primary endpoints (OS, MAE) depending on the number of GTMK present at the time of PHF diagnosis for the subgroups of sLPF & LPF (dark blue), RTSA (light blue) and non-operative treatment (gray). (s)LPF – locked plate fixation for simple and multi-fragment fracture, RTSA – reverse total shoulder arthroplasty

At two-year follow-up, the number of GTMK remained strongly associated with adverse outcomes across all examined endpoints. Increasing GTMK burden was consistently correlated with higher 2-year event rates for mortality, MAE, TE, SC, and MOC. As observed in the 1-year analysis, non-operative treatment was associated with higher 2-year mortality rates across nearly all GTMK strata compared to RTSA and LPF, with the differences being most pronounced in patients with moderate frailty burden (approximately 4–6 GTMK). For MAE and TE, event rates continued to increase in parallel with rising GTMK counts, with all treatment groups showing substantially elevated risks beyond 5 GTMK. Minor outpatient complications demonstrated an early and marked increase with even low GTMK counts, with rates exceeding 20% already at 2–3 GTMK. In contrast, surgical complications and mortality showed a more progressive risk increase, becoming particularly pronounced in patients with ≥ 6 GTMK. Across all endpoints, the protective effect of surgical treatment persisted but diminished as frailty burden intensified, with event rates converging across treatment groups in patients harboring very high GTMK counts (> 8). For further details refer to Fig. 4.

## Discussion

The key findings of this study demonstrate that specific individual GTMK domains were strong predictors of adverse outcomes. Malnutrition was a major predictor of mortality and TE, while decubital ulcers were strongly associated with MAE. The ongoing demographic shift toward an aging population continues to increase healthcare costs, largely driven by the growing proportion of frail patients who are at higher risk for fractures and complex injuries [4, 8, 11, 23, 24]. In response to this challenge, international organizations and healthcare alliances have highlighted the urgent need for reliable risk stratification models capable of identifying patients who are particularly vulnerable to frailty and its associated complications [25]. In orthopaedic and trauma surgery, this raises the fundamental question of whether surgical treatment is always the most appropriate option, especially in patients with a high frailty burden. To support these complex clinical decisions, multiple frailty and comorbidity-based scoring systems have been developed, including the mFI-5, the Hospital Frailty Risk Score, the Clinical Frailty Scale, and the Rockwood Frailty Index [3, 5, 9, 10, 17, 26–34]. Although these tools have demonstrated prognostic value in orthopaedic populations, important limitations remain. These include limited ability to predict long-term outcomes, restricted usefulness in guiding individualized decisions between surgical and non-surgical management, and insufficient representation of the multidimensional nature of frailty [35, 36]. The present study addresses these gaps by applying a more comprehensive and geriatric-specific frailty characterization, with the aim of improving long-term outcome prediction and better identifying which frail patients may still benefit from surgical treatment.

Within this framework, our results demonstrate that GTMKs are strong and durable predictors of mortality and MAE in geriatric patients with PHF. Several specific GTMK domains including malnutrition, decubital ulcers, cognitive impairment, and electrolyte disturbances emerged as particularly relevant contributors to long-term risk and should therefore be given special consideration during clinical decision-making. Surgical treatment remained beneficial in patients with low to moderate frailty burden; however, its advantage decreased as frailty accumulated, suggesting the presence of a threshold beyond which surgical intervention may no longer provide meaningful benefit. In summary, the GTMK framework offers a more nuanced, multidimensional, and geriatric-specific approach to risk stratification that may enhance existing frailty models and support more individualized treatment strategies. Importantly, this approach also facilitates early identification of modifiable risk factors, which may allow targeted interventions and potentially improve long-term outcomes.

In more detail, the cumulative number of GTMKs was strongly associated with increased risk across all examined endpoints, including overall survival, MAE, TE, SC. These findings are consistent with previous studies. For example, Yi et al. demonstrated that higher Modified Charlson Comorbidity Index and mFI-5 scores were associated with good predictive performance for short-term postoperative outcomes in patients treated for proximal humerus fractures [9] . Chan et al. reported that increasing Clinical Frailty Scale scores were associated with greater frailty and prolonged hospital length of stay. Our results align with this literature and extend our own previous work, which had already shown that GTMKs are predictive of short-term outcomes following proximal humerus fractures [37]. The present analysis expands these findings to long-term follow-up, demonstrating that GTMK burden remains a strong and independent predictor of adverse outcomes for up to five years after the initial diagnosis, regardless of treatment strategy.

Notably, mortality risk increased markedly from four GTMKs onward, while the risk for MAE increased from as few as two GTMKs. These patterns indicate that even relatively low levels of frailty may carry relevant prognostic information, depending on the specific endpoint considered. Similar observations have been reported by Jevadzadeh et al. using the Clinical Frailty Scale [38]. While mortality and MAE were mainly driven by advanced frailty accumulation, MOC appeared particularly sensitive to early frailty burden. In contrast to many existing frailty indices, which are primarily validated for short-term prediction horizons ranging from 30 days to one year, the GTMK framework demonstrated robust predictive performance across both short- and long-term follow-up periods. This allows a more comprehensive understanding of the sustained impact of multidimensional frailty on clinical outcomes in geriatric patients with PHF [15, 17].

Several individual GTMK domains emerged as particularly strong predictors across multiple endpoints, including overall survival, MAE, and TE. Malnutrition, decubital ulcers, cognitive impairment, and fluid and electrolyte disorders were consistently associated with substantially increased risk, identifying these domains as especially high-risk components within the GTMK framework. Some of these aspects are partially captured in existing scores such as the Clinical Frailty Scale Clinical Frailty Scale, Hospital Frailty Risk Score, or the Elixhauser Comorbidity Index. For example, Church et al. demonstrated that falls (71%), cognitive impairment (94%), and functional decline (91%) were the strongest predictors of mortality within the Clinical Frailty Scale [39]. The identification of these high-risk domains may offer opportunities for focused geriatric intervention and optimization prior to or alongside surgical treatment.

In contrast, certain GTMKs (including pain, sensory disorders, and visual or hearing impairment) were unexpectedly associated with lower risk across several outcomes. These findings should be interpreted with caution and may reflect selection bias, reverse causation, or residual confounding related to coding practices and data capture.

Surgical treatment, including RTSA and LPF, was generally associated with lower adverse event rates compared with non-operative management, particularly in patients with low to moderate frailty burden (4–6 GTMKs). However, as frailty burden increased, the protective effect of surgery diminished. At very high frailty levels (> 8 GTMKs), outcomes were comparable across treatment strategies, suggesting a ceiling effect in which frailty outweighs the potential benefits of surgical intervention. Compared with commonly used indices such as the mFI-5, which primarily reflect comorbidity, the GTMK framework offers a clear advantage by incorporating cognitive, functional, nutritional, and physiological domains. This multidimensional approach enables more comprehensive risk stratification beyond the perioperative period and remains predictive for long-term outcomes. The persistence of these associations at both one- and two-year follow-up highlights the durable prognostic value of GTMKs and supports their potential role in guiding individualized treatment decisions in this vulnerable patient population.

### Limitations

This retrospective study is subject to several limitations that should be reported. As with all retrospective analyses, potential biases and unmeasured confounding factors may influence the observed associations. One important limitation is the absence of detailed clinical information on fracture severity, displacement, or fracture classification, all of which may substantially impact recovery trajectories and complication rates following proximal humeral fracture. Furthermore, cognitive deficits were only identified through diagnostic coding and were not directly quantified in terms of severity or functional impairment. A fundamental constraint arises from the nature of the underlying data source: as administrative billing data were originally collected for reimbursement purposes rather than for research, certain clinical variables may be underrepresented or incompletely captured. This includes potential underreporting of frailty-related domains that are less frequently coded for billing but nonetheless clinically relevant. As such, the true prevalence of some geriatric-typical characteristic complexes may be underestimated, which could attenuate the observed associations in this analysis.

In conclusion, the GTMK framework provides a robust and multidimensional approach to frailty assessment that enables durable long-term risk stratification in geriatric patients with proximal humeral fractures. Its ability to capture nuanced vulnerabilities beyond conventional comorbidity-based scores offers valuable support for more individualized treatment decisions, particularly when weighing the risks and benefits of surgical intervention in this highly vulnerable population.

## Supplementary Information

Below is the link to the electronic supplementary material.

Supplementary Material 1

## Data Availability

The authors confirm that the data used in this study cannot be made available in the manuscript, in the supplementary files or in a public repository due to the Federal Data Protection Act (BDSG). They are stored on a BARMER server to facilitate replication of the results. In general access to statutory health insurance data for research purposes is only possible under the conditions laid down in the German Social Code (SGB V § 287).
