# Supplementary material for: The importance of comprehensive geriatric assessment in predicting the outcome of patients with proximal humerus fractures
Source: Aging Clin Exp Res. 2026 Mar 5;38(1):102. doi: 10.1007/s40520-026-03357-9 (PMC13005808; doi:10.1007/s40520-026-03357-9)
Supplement: Supplementary file 1 — Supplementary Material 1 [file 40520_2026_3357_MOESM1_ESM.docx]

**Fundamentals**

*Data Availability*

**Data availability:** The authors confirm that the data used in this study cannot be made available in the manuscript. in the supplementary files or in a public repository due to the Federal Data Protection Act (BDSG). They are stored on a BARMER server to facilitate replication of the results. In general. access to statutory health insurance data for research purposes is only possible under the conditions laid down in the German Social Code (SGB V § 287).

*ICD-/OPS-Codes and further Information*

**Overview of information sources:** A more detailed population description inclusion and exclusion criteria, as well as all ICD, OPS and ATC codes used for all influencing variables and all endpoints can be found in the Short Study Protocol in the supplements by Katthagen et al.^1^. Coding of the geriatrics-typical characteristic complexes (GTMK) was based on the ‘Abgrenzungskriterien der Geriatrie V1.3’ ^2,3^. The following table contains the complete overview of the GTMK codes used. Data collection was based on outpatient and inpatient information in the period of two years before the index event (i.e. the first coded proximal humeral fracture [PHF] diagnosis, ICD S42.2), which was coded as the main or second diagnosis. Deviating or supplementary comments are marked in the table and explained in the footnotes.

| **Operative interventions** | | |
| --- | --- | --- |
| Angular stable plate osteosynthesis for **multi-fragmented fracture** (LPF) | OPS | **5-794.21, 5-794.k1** |
| Angular stable plate osteosynthesis for **single fragment fracture** (sLPF) | OPS | **5-793.k1, 5-793.31** |
| Reverse total shoulder arthroplasty (RTSA) | OPS | **5-824.21** |
| Other fracture fixation | OPS | **5-790.01**, 5-790.11, 5-790.21, 5-790.31, 5-790.41, 5-790.51, 5-790.61, 5-790.71, 5-790.81, 5-790.91, 5-790.d1, 5-790.m1, 5-790.n1, 5-790.p1, 5-790.x1, **5-793.11**, 5-793.21, 5-793.41, 5-793.51, 5-793.61, 5-793.71, 5-793.81, 5-793.91, 5-793.a1, 5-793.b1, 5-793.c1, 5-793.g1, 5-793.m1, 5-793.n1, 5-793.x1, **5-794.01**, 5-794.11,5-794.31, 5-794.41, 5-794.51, 5-794.61, 5-794.71, 5-794.81, 5-794.a1, 5-794.b1, 5-794.c1, 5-794.g1, 5-794.m1, 5-794.n1, 5-794.x1, **5-824.00**, 5-824.01, 5-824.0x, 5-824.20 |
| **Geriatrics-typical characteristic complexes (GTMK)** | | |
| Immobility | ICD | **M**96.8, M62.3, M62.5 |
| Propensity for falls | ICD | **H**81, H82, **R**26, R29.81, R42 |
| Cognitive deficits | ICD | **F**00, F01, F02, F04, F05, F06.7, F07 |
| Incontinence | ICD | **N**39.3, N39.4, **R**15, R32 |
| Decubital ulcers | ICD | **I**830, I832, **L**89, L97, L984 |
| Malnutrition and malnourishment | ICD | **E**41, E43, E44, **R**64 |
| Fluids and electrolytes disorders | ICD | **E**86, E87, **R**60 |
| Depression and anxiety disorders | ICD | **F**30, F31, F32, F33, F40, F41 |
| Pain | ICD | *Acute pain*  **F**45.40*^[[1]](#footnote-1)^*, **H**57.1^1^, H92.0^1^, **K**08.88^1^, K14.6^1^, **M**25.51^1^, M54^1^, **N**23^1^, N64.4^1^, **R**07.1^1^, R07.2^1^, R07.3^1^, R07.4^1^, R10^1^, R51^1^, R52.0^1^, R52.9^1^  *Chronic pain*  **F**62.80, **R**52.1, R52.2 |
| Sensory disorders | ICD | **G**50-G59, G60-G64, **R**20 |
| Frailty | ICD | **R**54 |
| Severe visual and hearing impairment | ICD | **H**25, H28, H52.4, H53, H54, H90, H91 |
| Medication problems | ICD | **X**49.9, **Y**57.9 (in combination with T36-T50) |
| High risk of complications | ICD | **I**48, **S**02^[[2]](#footnote-2)^, S12^2^, S22^2^, S32^2^, S42^2^, S52^2^, S62^2^, S72^2^, S72^2^, S92^2^, **T**79-T89, **Z**43, Z48, Z98,  **Z**99.2 (dialysis requirement) |
|  | OPS | 8-853^[[3]](#footnote-3)^, 8-854^3^, 8-855^3^, 8-857^3^ |
| Delayed convalescence | ICD | **Z**54 |

*Table S1: ICD- and OPS-Codes for the operative interventions and Geriatric-typical characteristic complexes.*

Figure S1: 1-year rates with 95% confidence interval determined using Kaplan-Meier estimates of the primary endpoints (SC, MOC, MAE, TE) depending on the number of GTMK present at the time of PHF diagnosis for the subgroups of sLPF & LPF (dark blue), RTSA (light blue) and non-operative treatment (grey). (s)LPF – locked plate fixation for simple and multi-fragment fracture, RTSA –reverse total shoulder arthroplasty

| 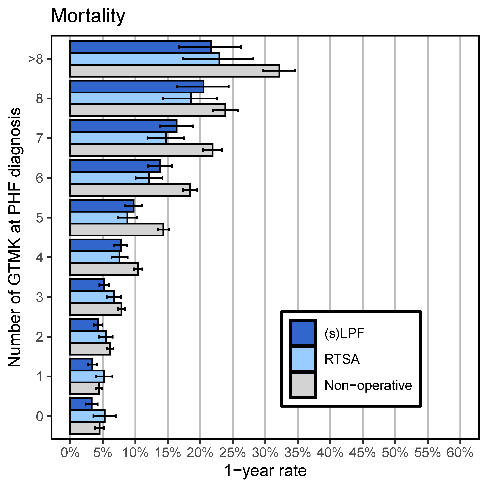 | 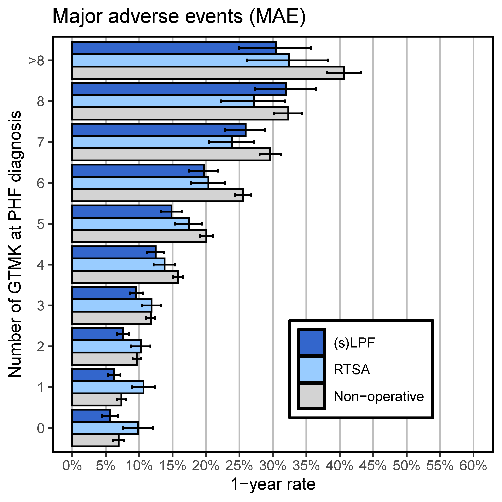 |
| --- | --- |
| 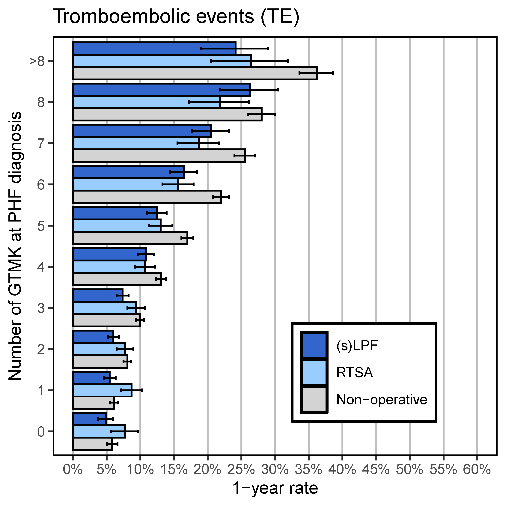 | 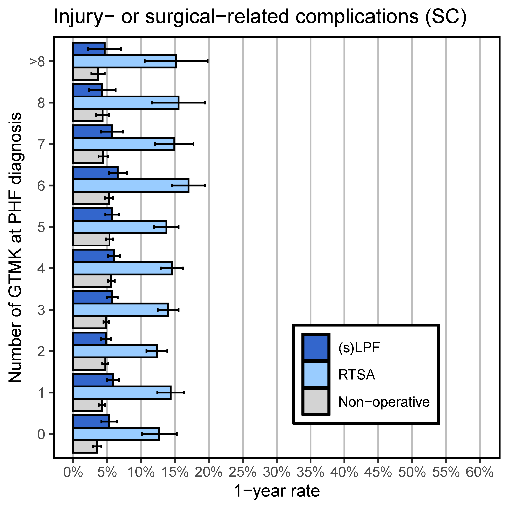 |
| 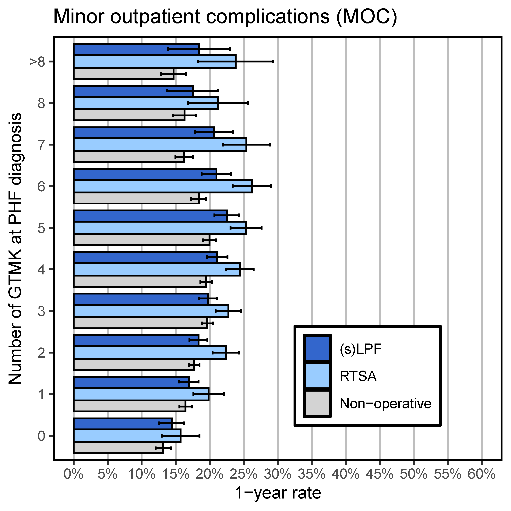 |  |

Figure S2: 2-year rates with 95% confidence interval determined using Kaplan-Meier estimates of the secondary endpoints (SC, MOC, TE) depending on the number of GTMK present at the time of PHF diagnosis for the subgroups of sLPF & LPF (dark blue), RTSA (light blue) and non-operative treatment (grey). (s)LPF – locked plate fixation for simple and multi-fragment fracture, RTSA –reverse total shoulder arthroplasty

| 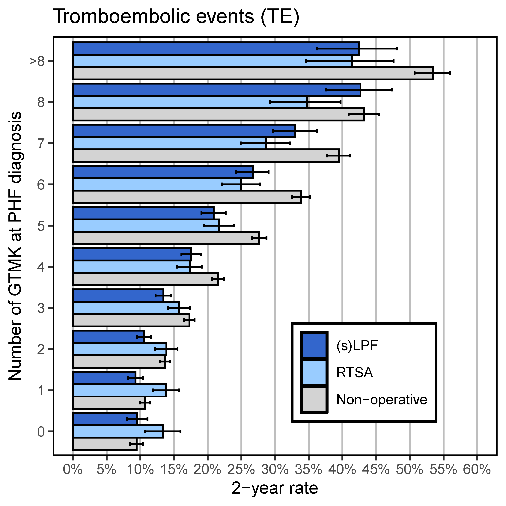 | 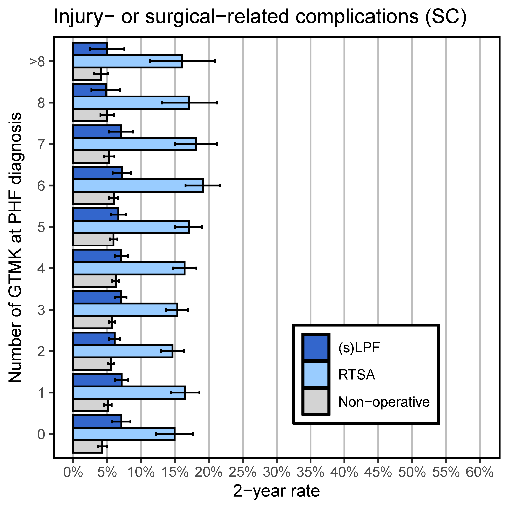 |
| --- | --- |
|  |  |
| 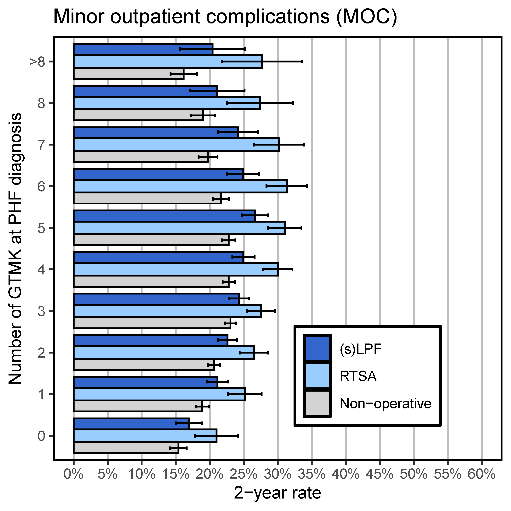 |  |

Figure S3: Kaplan-Meier estimates of the primary endpoints (SC, MOC, MAE, TE) stratified by the number of GTMK present at the time of PHF diagnosis. Curves represent all treatment groups combined and are color-coded from light green (no apparent GTMK) to dark blue (9 or more GTMK).

| 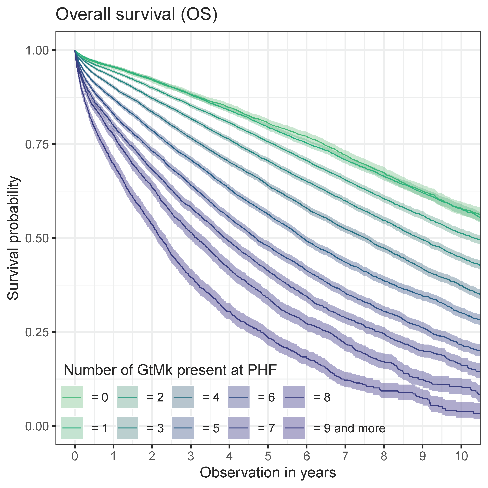 | 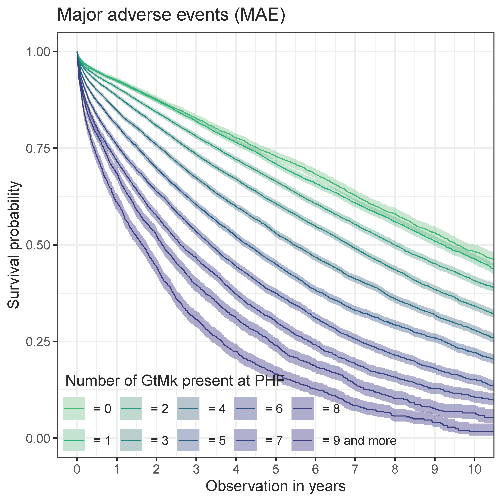 |
| --- | --- |
| 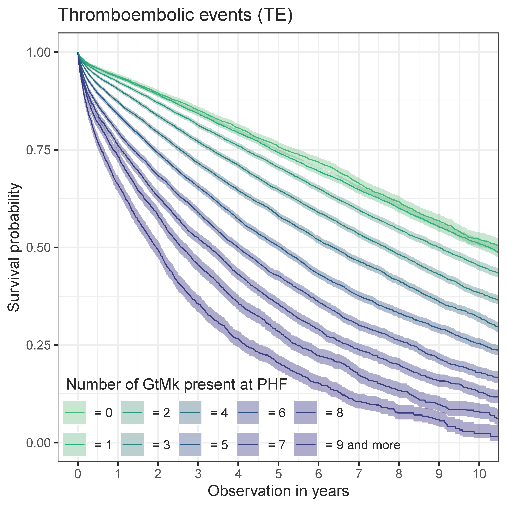 | 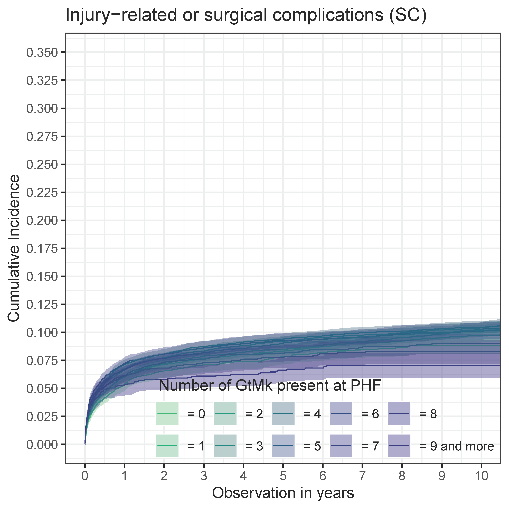 |
| 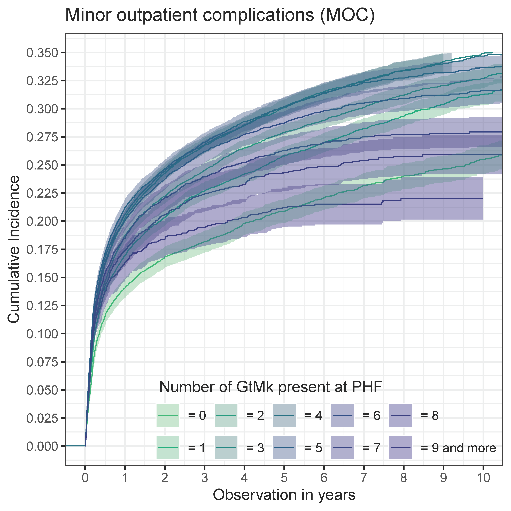 |  |

Table S2: 1-/2- and 5-year rates with 95% confidence interval (95%-CI) determined using Kaplan-Meier estimates of the primary endpoints (Mortality, SC, MOC, MAE, TE) depending on the number of GTMK present at the time of PHF diagnosis for the subgroups of (s)LPF, RTSA and non-operative treatment. For visualisation, see Figure 2 or Figure S2. (s)LPF – locked plate fixation for simple and multi-fragment fracture, RTSA –reverse total shoulder arthroplasty

| **Mortality** | | | | |
| --- | --- | --- | --- | --- |
|  | | **1-year rate  (95%-CI) (events)** | **2-years (95%-CI) (events)** | **5-years (95%-CI) (events)** |
| **(s)LPF** | No GTMK | 3.28% (2.35%, 4.20%) (47) | 6.56% (5.25%, 7.84%) (92) | 16.44% (14.34%, 18.49%) (207) |
|  | 1 GTMK | 3.38% (2.69%, 4.08%) (88) | 6.13% (5.19%, 7.06%) (156) | 18.00% (16.36%, 19.61%) (398) |
|  | 2 GTMK | 4.27% (3.59%, 4.96%) (143) | 7.66% (6.75%, 8.57%) (251) | 20.44% (18.94%, 21.92%) (592) |
|  | 3 GTMK | 5.23% (4.48%, 5.98%) (178) | 10.02% (8.99%, 11.04%) (333) | 24.20% (22.60%, 25.77%) (708) |
|  | 4 GTMK | 7.75% (6.74%, 8.75%) (212) | 13.33% (12.04%, 14.62%) (357) | 30.03% (28.10%, 31.90%) (709) |
|  | 5 GTMK | 9.75% (8.45%, 11.02%) (199) | 16.10% (14.47%, 17.70%) (322) | 38.46% (36.07%, 40.76%) (674) |
|  | 6 GTMK | 13.85% (11.98%, 15.67%) (186) | 23.08% (20.77%, 25.33%) (304) | 46.28% (43.24%, 49.16%) (544) |
|  | 7 GTMK | 16.37% (13.78%, 18.88%) (132) | 27.78% (24.58%, 30.85%) (219) | 54.65% (50.64%, 58.33%) (387) |
|  | 8 GTMK | 20.51% (16.45%, 24.37%) (82) | 36.83% (31.84%, 41.46%) (144) | 67.97% (62.43%, 72.70%) (242) |
|  | 9 and more | 21.65% (16.69%, 26.32%) (61) | 38.79% (32.69%, 44.34%) (106) | 72.10% (65.42%, 77.48%) (178) |
| **RTSA** | No GTMK | 5.29% (3.57%, 6.98%) (35) | 10.01% (7.64%, 12.31%) (64) | 28.60% (24.4%, 32.57%) (147) |
|  | 1 GTMK | 5.24% (3.99%, 6.47%) (65) | 9.09% (7.44%, 10.72%) (108) | 23.08% (20.24%, 25.82%) (221) |
|  | 2 GTMK | 5.45% (4.38%, 6.51%) (95) | 10.69% (9.19%, 12.17%) (178) | 25.75% (23.26%, 28.15%) (348) |
|  | 3 GTMK | 6.70% (5.58%, 7.81%) (129) | 11.88% (10.39%, 13.34%) (221) | 32.87% (30.26%, 35.38%) (478) |
|  | 4 GTMK | 7.58% (6.33%, 8.81%) (133) | 12.94% (11.32%, 14.54%) (219) | 35.20% (32.42%, 37.86%) (471) |
|  | 5 GTMK | 8.78% (7.29%, 10.25%) (124) | 16.51% (14.5%, 18.48%) (223) | 38.15% (35.04%, 41.11%) (422) |
|  | 6 GTMK | 12.14% (10.04%, 14.20%) (115) | 19.49% (16.85%, 22.04%) (177) | 44.5% (40.42%, 48.30%) (322) |
|  | 7 GTMK | 14.74% (11.89%, 17.51%) (90) | 23.17% (19.67%, 26.51%) (137) | 54.91% (49.75%, 59.54%) (261) |
|  | 8 GTMK | 18.52% (14.25%, 22.58%) (62) | 29.17% (24.03%, 33.97%) (95) | 59.52% (52.32%, 65.63%) (157) |
|  | 9 and more | 22.94% (17.33%, 28.18%) (53) | 36.48% (29.81%, 42.51%) (82) | 66.02% (57.29%, 72.96%) (122) |
| **Non-OP** | No GTMK | 4.51% (3.82%, 5.20%) (157) | 7.34% (6.46%, 8.21%) (250) | 18.24% (16.82%, 19.64%) (544) |
|  | 1 GTMK | 4.43% (3.93%, 4.92%) (292) | 7.78% (7.13%, 8.44%) (502) | 20.52% (19.44%, 21.59%) (1,150) |
|  | 2 GTMK | 6.16% (5.65%, 6.66%) (534) | 10.72% (10.05%, 11.37%) (907) | 25.07% (24.05%, 26.07%) (1,858) |
|  | 3 GTMK | 7.87% (7.32%, 8.41%) (736) | 13.77% (13.06%, 14.48%) (1,256) | 30.5% (29.46%, 31.52%) (2,449) |
|  | 4 GTMK | 10.44% (9.78%, 11.09%) (866) | 17.63% (16.79%, 18.46%) (1,428) | 37.77% (36.6%, 38.92%) (2,694) |
|  | 5 GTMK | 14.32% (13.49%, 15.15%) (978) | 23.55% (22.52%, 24.57%) (1,569) | 45.78% (44.43%, 47.09%) (2,697) |
|  | 6 GTMK | 18.47% (17.38%, 19.54%) (912) | 28.98% (27.69%, 30.26%) (1,399) | 54.82% (53.21%, 56.37%) (2,333) |
|  | 7 GTMK | 21.89% (20.44%, 23.32%) (691) | 34.35% (32.64%, 36.01%) (1,059) | 60.52% (58.53%, 62.42%) (1,667) |
|  | 8 GTMK | 23.86% (21.91%, 25.77%) (448) | 37.81% (35.54%, 39.99%) (695) | 65.77% (63.21%, 68.14%) (1,084) |
|  | 9 and more | 32.18% (29.71%, 34.56%) (458) | 49.14% (46.42%, 51.73%) (682) | 79.24% (76.57%, 81.61%) (991) |

| **Major Adverse Events (MAE)** | | | | |
| --- | --- | --- | --- | --- |
|  | | **1-year rate  (95%-CI) (events)** | **2-years (95%-CI) (events)** | **5-years (95%-CI) (events)** |
| **(s)LPF** | No GTMK | 5.65% (4.45%, 6.84%) (81) | 10.66% (9.03%, 12.26%) (150) | 23.46% (21.07%, 25.78%) (300) |
|  | 1 GTMK | 6.27% (5.33%, 7.19%) (163) | 10.35% (9.16%, 11.53%) (264) | 26.33% (24.44%, 28.17%) (589) |
|  | 2 GTMK | 7.62% (6.72%, 8.52%) (255) | 12.88% (11.73%, 14.02%) (423) | 29.91% (28.2%, 31.58%) (879) |
|  | 3 GTMK | 9.58% (8.59%, 10.56%) (326) | 16.32% (15.06%, 17.57%) (545) | 34.32% (32.54%, 36.04%) (1023) |
|  | 4 GTMK | 12.47% (11.22%, 13.7%) (341) | 20.48% (18.93%, 21.99%) (549) | 40.61% (38.53%, 42.61%) (972) |
|  | 5 GTMK | 14.84% (13.28%, 16.37%) (303) | 25.26% (23.32%, 27.14%) (505) | 49.39% (46.93%, 51.74%) (887) |
|  | 6 GTMK | 19.65% (17.5%, 21.75%) (264) | 30.84% (28.29%, 33.3%) (407) | 56.04% (52.99%, 58.9%) (668) |
|  | 7 GTMK | 25.92% (22.83%, 28.88%) (209) | 39.64% (36.12%, 42.97%) (314) | 66.2% (62.32%, 69.68%) (479) |
|  | 8 GTMK | 32.01% (27.28%, 36.44%) (128) | 47.41% (42.2%, 52.16%) (186) | 78.51% (73.47%, 82.58%) (284) |
|  | 9 and more | 30.52% (24.93%, 35.69%) (86) | 46.16% (39.88%, 51.78%) (127) | 77.14% (70.82%, 82.10%) (194) |
| **RTSA** | No GTMK | 9.82% (7.53%, 12.06%) (65) | 17.29% (14.30%, 20.17%) (111) | 38.35% (33.85%, 42.54%) (206) |
|  | 1 GTMK | 10.64% (8.91%, 12.34%) (132) | 17.27% (15.08%, 19.39%) (206) | 34.53% (31.34%, 37.56%) (347) |
|  | 2 GTMK | 10.27% (8.83%, 11.68%) (179) | 17.17% (15.34%, 18.96%) (289) | 36.55% (33.81%, 39.17%) (510) |
|  | 3 GTMK | 11.85% (10.39%, 13.28%) (228) | 18.95% (17.15%, 20.72%) (354) | 43.85% (41.12%, 46.46%) (668) |
|  | 4 GTMK | 13.79% (12.16%, 15.39%) (242) | 21.8% (19.80%, 23.75%) (370) | 48.71% (45.79%, 51.47%) (677) |
|  | 5 GTMK | 17.41% (15.41%, 19.37%) (246) | 27.28% (24.86%, 29.62%) (372) | 53.24% (50.01%, 56.26%) (613) |
|  | 6 GTMK | 20.37% (17.76%, 22.89%) (193) | 30.52% (27.45%, 33.46%) (280) | 58.68% (54.58%, 62.4%) (445) |
|  | 7 GTMK | 23.91% (20.45%, 27.22%) (146) | 35.82% (31.82%, 39.58%) (213) | 65.8% (60.93%, 70.07%) (336) |
|  | 8 GTMK | 27.18% (22.25%, 31.79%) (91) | 42.52% (36.82%, 47.7%) (138) | 69.48% (62.65%, 75.05%) (193) |
|  | 9 and more | 32.47% (26.15%, 38.24%) (75) | 52.62% (45.45%, 58.85%) (117) | 77.10% (69.14%, 83.01%) (151) |
| **Non-OP** | No GTMK | 6.93% (6.08%, 7.77%) (241) | 11.15% (10.09%, 12.20%) (380) | 25.5% (23.89%, 27.07%) (769) |
|  | 1 GTMK | 7.35% (6.72%, 7.98%) (485) | 12.33% (11.52%, 13.13%) (796) | 28.81% (27.59%, 30.00%) (1,636) |
|  | 2 GTMK | 9.63% (9%, 10.25%) (835) | 16.04% (15.25%, 16.81%) (1,359) | 33.73% (32.62%, 34.81%) (2,540) |
|  | 3 GTMK | 11.73% (11.08%, 12.38%) (1,098) | 19.98% (19.15%, 20.80%) (1,824) | 40.11% (39%, 41.19%) (3,271) |
|  | 4 GTMK | 15.81% (15.02%, 16.59%) (1,312) | 25.06% (24.11%, 26.00%) (2,036) | 48.34% (47.13%, 49.53%) (3,503) |
|  | 5 GTMK | 20.06% (19.10%, 21.00%) (1,370) | 31.76% (30.63%, 32.87%) (2,120) | 56.08% (54.73%, 57.39%) (3,355) |
|  | 6 GTMK | 25.57% (24.34%, 26.78%) (1,263) | 38.36% (36.97%, 39.72%) (1,855) | 64.73% (63.18%, 66.22%) (2,812) |
|  | 7 GTMK | 29.62% (28.01%, 31.19%) (935) | 44.04% (42.25%, 45.77%) (1,361) | 71.04% (69.17%, 72.80%) (1,992) |
|  | 8 GTMK | 32.32% (30.17%, 34.40%) (607) | 48.76% (46.41%, 51.01%) (899) | 75.78% (73.43%, 77.92%) (1,275) |
|  | 9 and more | 40.68% (38.07%, 43.17%) (579) | 57.54% (54.83%, 60.08%) (802) | 85.33% (82.94%, 87.39%) (1,087) |

| **Thromboembolic Events (TE)** | | | | |
| --- | --- | --- | --- | --- |
|  | | **1-year rate  (95%-CI) (events)** | **2-years (95%-CI) (events)** | **5-years (95%-CI) (events)** |
| **(s)LPF** | No GTMK | 4.88% (3.76%, 5.99%) (70) | 9.54% (7.99%, 11.07%) (134) | 21.30% (18.99%, 23.55%) (271) |
|  | 1 GTMK | 5.46% (4.58%, 6.33%) (142) | 9.30% (8.16%, 10.42%) (237) | 23.27% (21.47%, 25.03%) (521) |
|  | 2 GTMK | 6.01% (5.20%, 6.81%) (201) | 10.55% (9.49%, 11.60%) (346) | 26.59% (24.93%, 28.20%) (775) |
|  | 3 GTMK | 7.38% (6.49%, 8.25%) (251) | 13.43% (12.26%, 14.58%) (447) | 29.55% (27.84%, 31.21%) (874) |
|  | 4 GTMK | 10.86% (9.68%, 12.02%) (297) | 17.56% (16.10%, 18.99%) (471) | 35.96% (33.95%, 37.92%) (859) |
|  | 5 GTMK | 12.49% (11.04%, 13.91%) (255) | 20.90% (19.09%, 22.66%) (418) | 44.59% (42.14%, 46.93%) (792) |
|  | 6 GTMK | 16.45% (14.44%, 18.41%) (221) | 26.69% (24.26%, 29.05%) (352) | 50.52% (47.47%, 53.39%) (600) |
|  | 7 GTMK | 20.46% (17.63%, 23.20%) (165) | 33.02% (29.65%, 36.23%) (261) | 60.59% (56.62%, 64.20%) (433) |
|  | 8 GTMK | 26.26% (21.82%, 30.45%) (105) | 42.68% (37.53%, 47.40%) (167) | 73.07% (67.68%, 77.56%) (261) |
|  | 9 and more | 24.14% (18.97%, 28.97%) (68) | 42.48% (36.25%, 48.09%) (116) | 76.00% (69.51%, 81.10%) (188) |
| **RTSA** | No GTMK | 7.71% (5.65%, 9.72%) (51) | 13.36% (10.68%, 15.95%) (86) | 34.24% (29.83%, 38.37%) (180) |
|  | 1 GTMK | 8.71% (7.12%, 10.26%) (108) | 13.84% (11.85%, 15.78%) (165) | 30.04% (26.95%, 33.00%) (295) |
|  | 2 GTMK | 7.74% (6.48%, 8.99%) (135) | 13.88% (12.20%, 15.53%) (232) | 30.79% (28.17%, 33.32%) (423) |
|  | 3 GTMK | 9.41% (8.09%, 10.70%) (181) | 15.74% (14.06%, 17.38%) (293) | 39.78% (37.05%, 42.38%) (589) |
|  | 4 GTMK | 10.71% (9.25%, 12.15%) (188) | 17.32% (15.49%, 19.11%) (294) | 42.45% (39.58%, 45.19%) (581) |
|  | 5 GTMK | 13.03% (11.26%, 14.77%) (184) | 21.71% (19.48%, 23.89%) (295) | 45.55% (42.34%, 48.58%) (513) |
|  | 6 GTMK | 15.62% (13.28%, 17.91%) (148) | 24.97% (22.08%, 27.75%) (227) | 51.03% (46.89%, 54.85%) (377) |
|  | 7 GTMK | 18.67% (15.52%, 21.71%) (114) | 28.64% (24.89%, 32.20%) (170) | 61.11% (56.06%, 65.58%) (300) |
|  | 8 GTMK | 21.81% (17.26%, 26.11%) (73) | 34.70% (29.29%, 39.71%) (113) | 64.84% (57.75%, 70.74%) (175) |
|  | 9 and more | 26.41% (20.5%, 31.88%) (61) | 41.44% (34.56%, 47.59%) (93) | 70.83% (62.34%, 77.41%) (133) |
| **Non-OP** | No GTMK | 5.84% (5.05%, 6.61%) (203) | 9.46% (8.47%, 10.44%) (322) | 23.05% (21.49%, 24.57%) (689) |
|  | 1 GTMK | 6.11% (5.53%, 6.68%) (403) | 10.69% (9.93%, 11.44%) (689) | 25.57% (24.40%, 26.72%) (1,447) |
|  | 2 GTMK | 8.08% (7.51%, 8.65%) (701) | 13.65% (12.91%, 14.38%) (1,156) | 30.06% (28.98%, 31.12%) (2,247) |
|  | 3 GTMK | 9.97% (9.36%, 10.58%) (933) | 17.29% (16.50%, 18.06%) (1,577) | 36.27% (35.18%, 37.34%) (2,936) |
|  | 4 GTMK | 13.05% (12.32%, 13.77%) (1,083) | 21.53% (20.63%, 22.42%) (1,746) | 43.49% (42.29%, 44.67%) (3,127) |
|  | 5 GTMK | 17.00% (16.10%, 17.89%) (1,161) | 27.66% (26.57%, 28.73%) (1,843) | 51.35% (50.00%, 52.67%) (3,045) |
|  | 6 GTMK | 21.99% (20.82%, 23.13%) (1,086) | 33.87% (32.52%, 35.20%) (1,636) | 60.00% (58.41%, 61.53%) (2,581) |
|  | 7 GTMK | 25.5% (23.97%, 27.01%) (805) | 39.46% (37.70%, 41.17%) (1,217) | 66.31% (64.37%, 68.14%) (1,842) |
|  | 8 GTMK | 28.01% (25.95%, 30.01%) (526) | 43.25% (40.92%, 45.48%) (796) | 71.87% (69.42%, 74.12%) (1,194) |
|  | 9 and more | 36.18% (33.64%, 38.63%) (515) | 53.43% (50.70%, 56.01%) (742) | 81.77% (79.21%, 84.03%) (1,031) |

| **Injury- or Surgery-related Complications (SC)** | | | | |
| --- | --- | --- | --- | --- |
|  | | **1-year rate  (95%-CI) (events)** | **2-years (95%-CI) (events)** | **5-years (95%-CI) (events)** |
| **(s)LPF** | No GTMK | 5.30% (4.14%, 6.46%) (76) | 7.10% (5.76%, 8.43%) (101) | 7.27% (5.91%, 8.62%) (103) |
|  | 1 GTMK | 5.88% (4.98%, 6.79%) (153) | 7.12% (6.12%, 8.11%) (184) | 7.58% (6.55%, 8.61%) (194) |
|  | 2 GTMK | 4.81% (4.09%, 5.54%) (161) | 6.11% (5.29%, 6.92%) (203) | 6.55% (5.70%, 7.40%) (216) |
|  | 3 GTMK | 5.76% (4.98%, 6.54%) (196) | 6.98% (6.12%, 7.84%) (236) | 7.79% (6.88%, 8.71%) (259) |
|  | 4 GTMK | 6.03% (5.14%, 6.93%) (165) | 7.06% (6.10%, 8.03%) (192) | 7.88% (6.85%, 8.90%) (210) |
|  | 5 GTMK | 5.73% (4.72%, 6.74%) (117) | 6.64% (5.56%, 7.72%) (135) | 7.22% (6.08%, 8.36%) (144) |
|  | 6 GTMK | 6.55% (5.22%, 7.87%) (88) | 7.16% (5.78%, 8.55%) (96) | 7.79% (6.34%, 9.24%) (103) |
|  | 7 GTMK | 5.70% (4.10%, 7.30%) (46) | 7.00% (5.23%, 8.77%) (56) | 7.78% (5.90%, 9.67%) (61) |
|  | 8 GTMK | 4.25% (2.27%, 6.24%) (17) | 4.77% (2.67%, 6.87%) (19) | 5.34% (3.11%, 7.56%) (21) |
|  | 9 and more | 4.61% (2.16%, 7.07%) (13) | 4.98% (2.43%, 7.54%) (14) | 5.46% (2.75%, 8.16%) (15) |
| **RTSA** | No GTMK | 12.70% (10.15%, 15.24%) (84) | 14.94% (12.20%, 17.67%) (98) | 16.94% (13.99%, 19.89%) (108) |
|  | 1 GTMK | 14.34% (12.39%, 16.30%) (178) | 16.51% (14.43%, 18.59%) (203) | 19.50% (17.15%, 21.85%) (226) |
|  | 2 GTMK | 12.33% (10.78%, 13.87%) (215) | 14.63% (12.96%, 16.31%) (252) | 17.03% (15.19%, 18.87%) (282) |
|  | 3 GTMK | 13.97% (12.42%, 15.52%) (269) | 15.34% (13.72%, 16.96%) (294) | 17.70% (15.93%, 19.47%) (326) |
|  | 4 GTMK | 14.53% (12.88%, 16.18%) (255) | 16.41% (14.66%, 18.15%) (285) | 18.45% (16.56%, 20.34%) (309) |
|  | 5 GTMK | 13.73% (11.93%, 15.52%) (194) | 17.03% (15.05%, 19.01%) (237) | 20.02% (17.80%, 22.24%) (264) |
|  | 6 GTMK | 16.99% (14.59%, 19.38%) (161) | 19.08% (16.55%, 21.60%) (179) | 23.08% (20.14%, 26.02%) (200) |
|  | 7 GTMK | 14.90% (12.07%, 17.73%) (91) | 18.10% (15.02%, 21.19%) (109) | 19.76% (16.50%, 23.03%) (116) |
|  | 8 GTMK | 15.53% (11.64%, 19.42%) (52) | 17.11% (13.05%, 21.17%) (57) | 19.23% (14.73%, 23.73%) (61) |
|  | 9 and more | 15.15% (10.51%, 19.79%) (35) | 16.09% (11.32%, 20.85%) (37) | 18.69% (13.21%, 24.17%) (40) |
| **Non-OP** | No GTMK | 3.53% (2.92%, 4.15%) (123) | 4.29% (3.61%, 4.96%) (148) | 6.01% (5.18%, 6.84%) (194) |
|  | 1 GTMK | 4.21% (3.72%, 4.69%) (278) | 5.11% (4.57%, 5.64%) (334) | 6.36% (5.76%, 6.97%) (399) |
|  | 2 GTMK | 4.72% (4.28%, 5.17%) (410) | 5.55% (5.06%, 6.03%) (478) | 7.15% (6.58%, 7.71%) (587) |
|  | 3 GTMK | 4.88% (4.44%, 5.32%) (457) | 5.72% (5.25%, 6.20%) (532) | 7.45% (6.89%, 8.00%) (655) |
|  | 4 GTMK | 5.59% (5.09%, 6.08%) (464) | 6.27% (5.75%, 6.80%) (518) | 7.73% (7.14%, 8.33%) (612) |
|  | 5 GTMK | 5.33% (4.79%, 5.86%) (364) | 5.93% (5.37%, 6.50%) (403) | 7.41% (6.76%, 8.06%) (477) |
|  | 6 GTMK | 5.24% (4.62%, 5.86%) (259) | 5.95% (5.29%, 6.61%) (292) | 7.05% (6.31%, 7.78%) (333) |
|  | 7 GTMK | 4.43% (3.72%, 5.15%) (140) | 5.28% (4.50%, 6.07%) (165) | 6.02% (5.16%, 6.87%) (182) |
|  | 8 GTMK | 4.31% (3.39%, 5.23%) (81) | 4.98% (3.99%, 5.97%) (93) | 5.94% (4.83%, 7.05%) (106) |
|  | 9 and more | 3.65% (2.68%, 4.63%) (52) | 4.09% (3.06%, 5.12%) (58) | 4.96% (3.77%, 6.15%) (66) |

| **Minor Outpatient Complications (MOC)** | | | | |
| --- | --- | --- | --- | --- |
|  | | **1-year rate  (95%-CI) (events)** | **2-years (95%-CI) (events)** | **5-years (95%-CI) (events)** |
| **(s)LPF** | No GTMK | 14.43% (12.61%, 16.25%) (207) | 16.96% (15.01%, 18.91%) (242) | 20.24% (18.11%, 22.37%) (281) |
|  | 1 GTMK | 16.91% (15.47%, 18.35%) (440) | 21.14% (19.57%, 22.72%) (546) | 26.69% (24.93%, 28.45%) (664) |
|  | 2 GTMK | 18.34% (17.03%, 19.66%) (614) | 22.61% (21.18%, 24.03%) (751) | 28.86% (27.27%, 30.45%) (923) |
|  | 3 GTMK | 19.72% (18.38%, 21.06%) (671) | 24.33% (22.88%, 25.78%) (821) | 30.57% (28.96%, 32.17%) (993) |
|  | 4 GTMK | 21.09% (19.56%, 22.62%) (577) | 24.95% (23.32%, 26.58%) (678) | 31.80% (29.97%, 33.62%) (825) |
|  | 5 GTMK | 22.51% (20.70%, 24.33%) (460) | 26.62% (24.69%, 28.55%) (540) | 32.28% (30.16%, 34.39%) (629) |
|  | 6 GTMK | 20.98% (18.80%, 23.16%) (282) | 24.90% (22.57%, 27.22%) (333) | 31.43% (28.84%, 34.01%) (402) |
|  | 7 GTMK | 20.67% (17.87%, 23.46%) (167) | 24.15% (21.18%, 27.12%) (194) | 29.42% (26.13%, 32.71%) (226) |
|  | 8 GTMK | 17.50% (13.77%, 21.24%) (70) | 21.10% (17.08%, 25.12%) (84) | 24.32% (20.03%, 28.61%) (95) |
|  | 9 and more | 18.45% (13.91%, 22.99%) (52) | 20.41% (15.66%, 25.16%) (57) | 22.62% (17.61%, 27.62%) (62) |
| **RTSA** | No GTMK | 15.71% (12.94%, 18.49%) (104) | 21.01% (17.87%, 24.15%) (137) | 23.49% (20.15%, 26.84%) (149) |
|  | 1 GTMK | 19.83% (17.61%, 22.05%) (246) | 25.17% (22.73%, 27.62%) (307) | 32.07% (29.25%, 34.89%) (364) |
|  | 2 GTMK | 22.36% (20.40%, 24.32%) (390) | 26.47% (24.38%, 28.57%) (456) | 32.96% (30.58%, 35.33%) (532) |
|  | 3 GTMK | 22.75% (20.87%, 24.62%) (438) | 27.56% (25.54%, 29.57%) (524) | 33.67% (31.41%, 35.93%) (604) |
|  | 4 GTMK | 24.45% (22.43%, 26.46%) (429) | 29.99% (27.82%, 32.16%) (518) | 36.59% (34.16%, 39.03%) (594) |
|  | 5 GTMK | 25.33% (23.06%, 27.61%) (358) | 31.02% (28.58%, 33.47%) (431) | 40.38% (37.55%, 43.20%) (518) |
|  | 6 GTMK | 26.17% (23.37%, 28.98%) (248) | 31.33% (28.35%, 34.32%) (293) | 38.78% (35.40%, 42.16%) (339) |
|  | 7 GTMK | 25.38% (21.92%, 28.84%) (155) | 30.15% (26.47%, 33.83%) (182) | 36.11% (32.02%, 40.19%) (206) |
|  | 8 GTMK | 21.21% (16.82%, 25.60%) (71) | 27.41% (22.55%, 32.26%) (90) | 32.10% (26.61%, 37.58%) (99) |
|  | 9 and more | 23.81% (18.30%, 29.33%) (55) | 27.71% (21.84%, 33.58%) (63) | 31.78% (25.22%, 38.35%) (68) |
| **Non-OP** | No GTMK | 13.16% (12.03%, 14.28%) (458) | 15.41% (14.20%, 16.62%) (532) | 19.70% (18.32%, 21.08%) (648) |
|  | 1 GTMK | 16.45% (15.55%, 17.35%) (1,086) | 18.91% (17.96%, 19.86%) (1,240) | 23.99% (22.92%, 25.07%) (1,501) |
|  | 2 GTMK | 17.70% (16.90%, 18.51%) (1,536) | 20.63% (19.78%, 21.49%) (1,777) | 25.90% (24.94%, 26.86%) (2,133) |
|  | 3 GTMK | 19.62% (18.82%, 20.43%) (1,837) | 23.04% (22.19%, 23.90%) (2,140) | 28.61% (27.65%, 29.57%) (2,542) |
|  | 4 GTMK | 19.48% (18.62%, 20.33%) (1,617) | 22.79% (21.88%, 23.7%) (1,876) | 28.19% (27.18%, 29.20%) (2,217) |
|  | 5 GTMK | 19.98% (19.03%, 20.93%) (1,365) | 22.79% (21.79%, 23.79%) (1,546) | 27.67% (26.57%, 28.77%) (1,801) |
|  | 6 GTMK | 18.38% (17.30%, 19.46%) (908) | 21.68% (20.52%, 22.83%) (1,060) | 26.13% (24.86%, 27.41%) (1,226) |
|  | 7 GTMK | 16.25% (14.96%, 17.53%) (513) | 19.75% (18.35%, 21.15%) (617) | 23.32% (21.79%, 24.86%) (700) |
|  | 8 GTMK | 16.29% (14.62%, 17.96%) (306) | 19.01% (17.22%, 20.79%) (354) | 22.83% (20.85%, 24.81%) (407) |
|  | 9 and more | 14.68% (12.84%, 16.52%) (209) | 16.16% (14.24%, 18.09%) (229) | 18.88% (16.76%, 20.99%) (256) |

|  |
| --- |
|  |
| 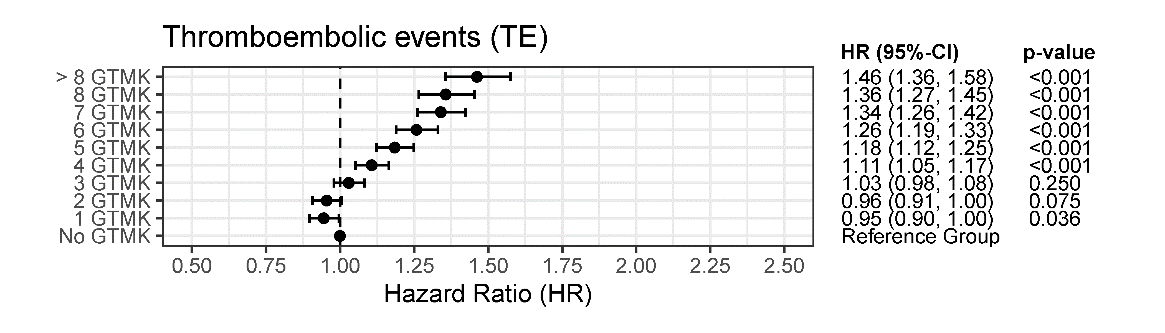  Figure S4: Influence of the number of GTMK present at the time of PHF on the risk of experiencing a primary event, among patients who did not experience any event within the first 3 months. Hazard ratios (HR) were adjusted for patients’ risk profiles using multivariable Cox regression, making these HR estimates independent of the treatment group. Complete regression results are provided in Table S3 to Table S7. |
| 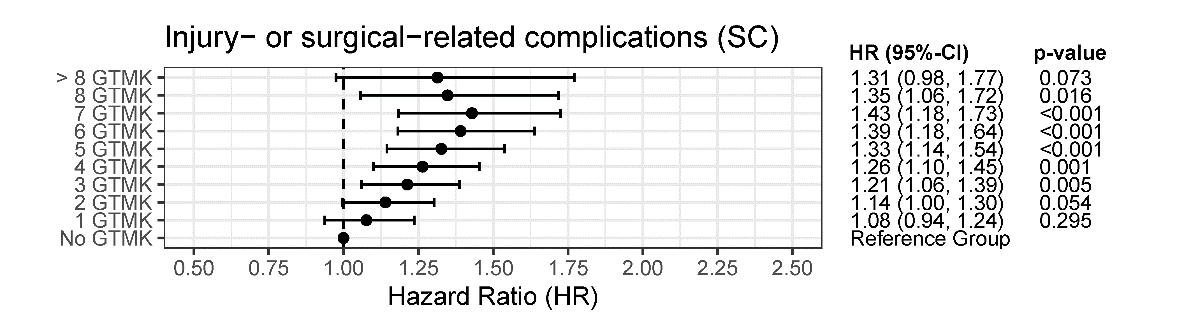 |
| 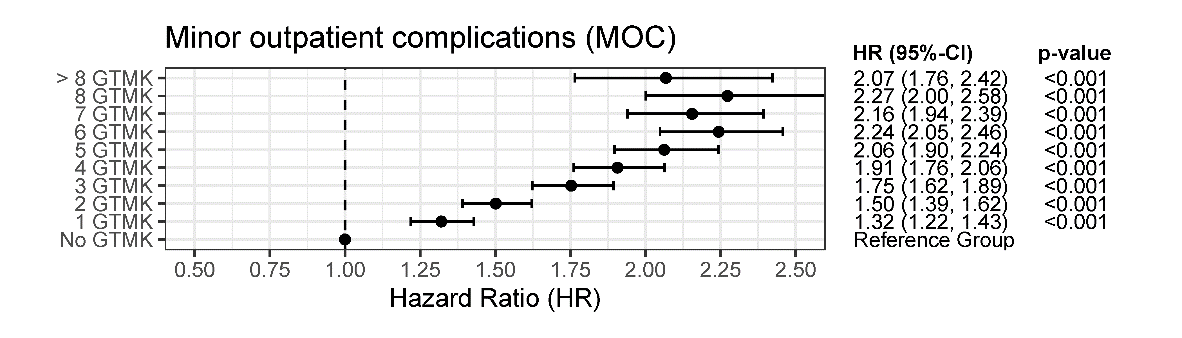 |

Figure S5: Influence of individual GTMK on Major Adverse Events (MAE), adjusted for patient risk profile. The analysis was performed separately for non-operative patients (grey), operative patients (blue), and the overall cohort (black), including only patients who remained event-free after 3 months. Interaction p-values (p_int) were calculated using a Cox regression model with interaction terms between each GTMK and the binary surgery variable, measuring differences in GTMK effects between non-operative and operative subgroups. Complete regression results are provided in Table S9.


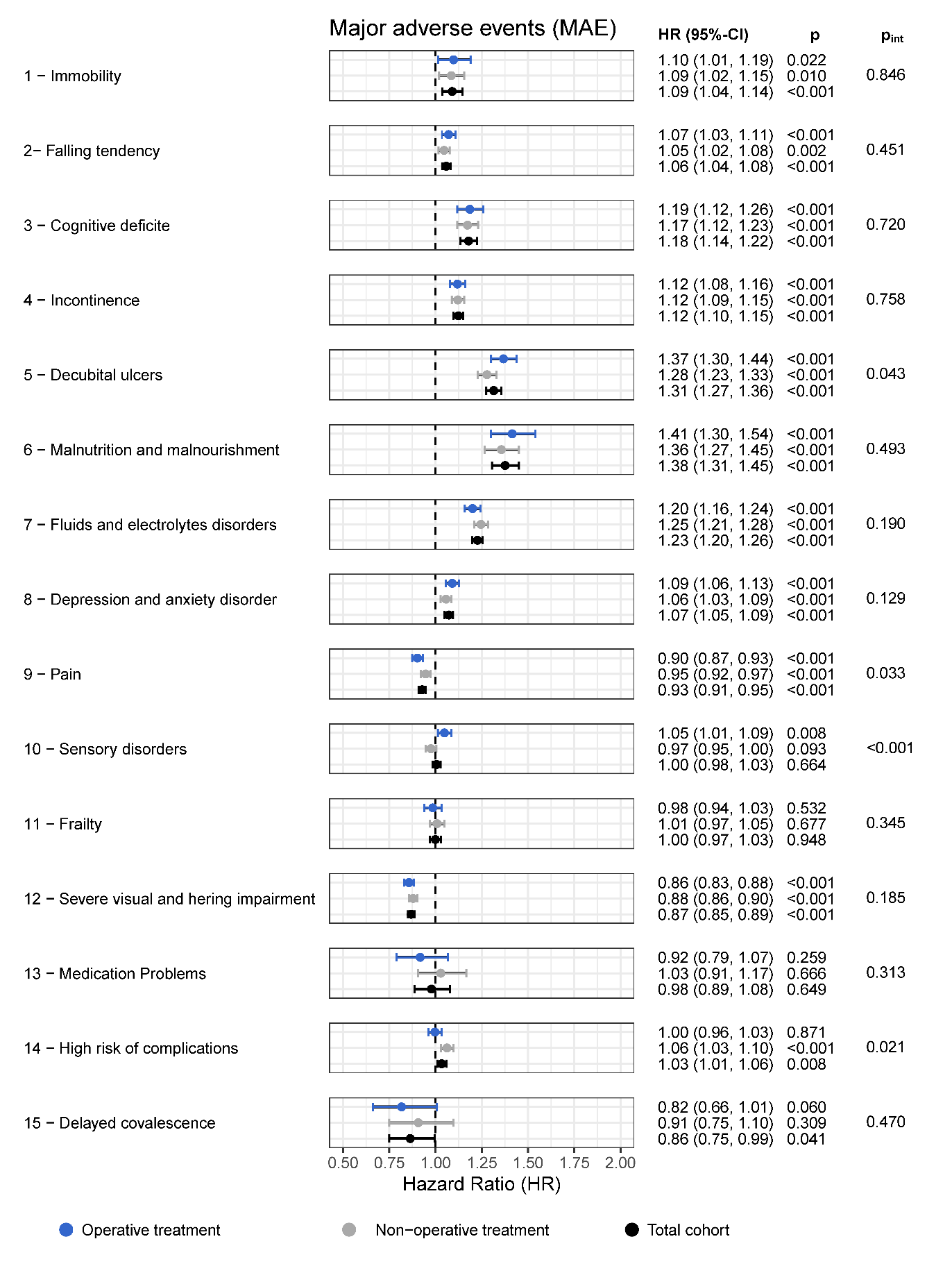


Figure S6: Influence of individual GTMK on Thromboembolic Events (TE), adjusted for patient risk profile. The analysis was performed separately for non-operative patients (grey), operative patients (blue), and the overall cohort (black), including only patients who remained event-free after 3 months. Interaction p-values (p_int) were calculated using a Cox regression model with interaction terms between each GTMK and the binary surgery variable, measuring differences in GTMK effects between non-operative and operative subgroups. Complete regression results are provided in Table S10.


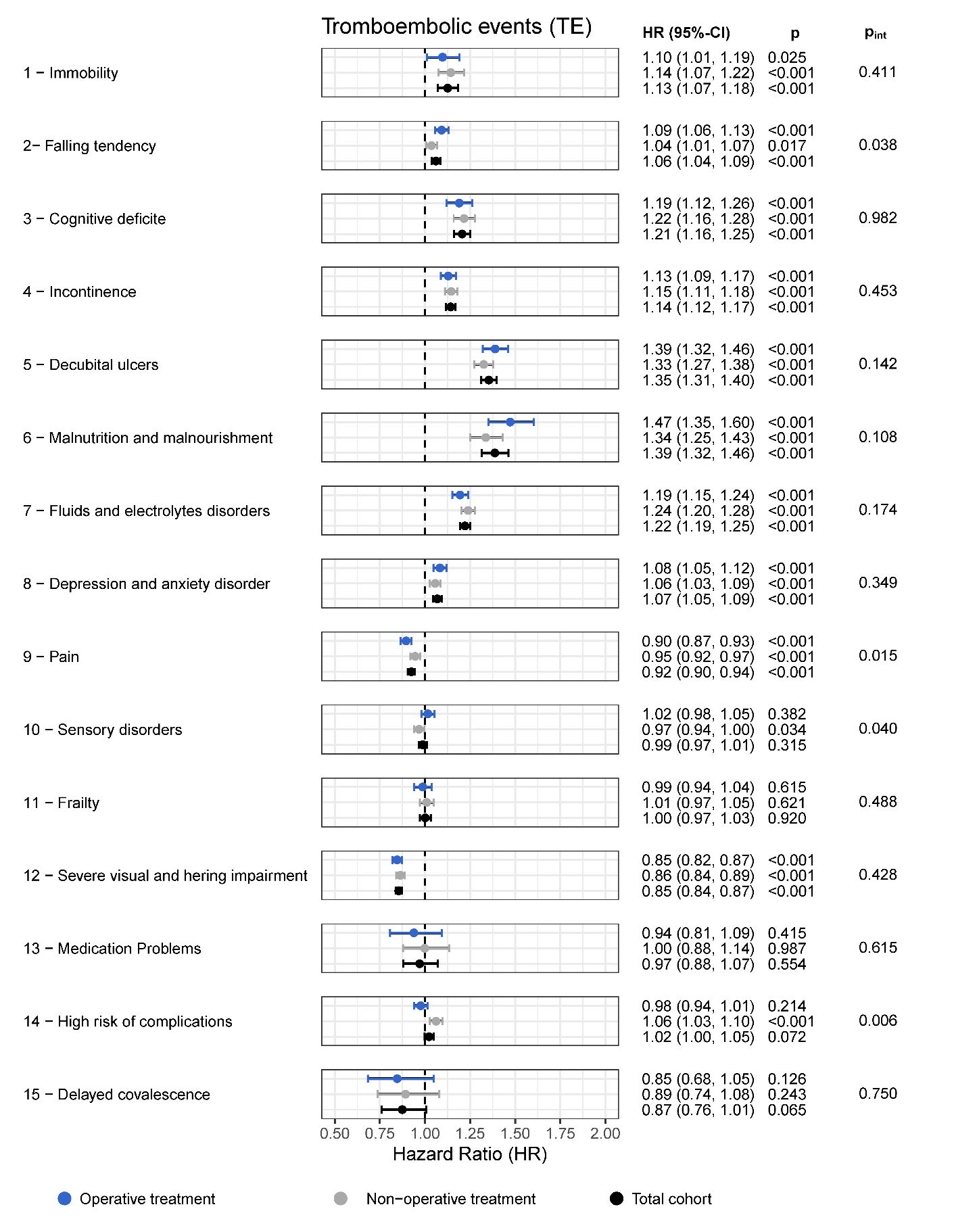


Figure S7: Influence of individual GTMK on Injury- or Operative-related Complications (SC), adjusted for patient risk profile. The analysis was performed separately for non-operative patients (grey), operative patients (blue), and the overall cohort (black), including only patients who remained event-free after 3 months. Interaction p-values (p_int) were calculated using a Cox regression model with interaction terms between each GTMK and the binary surgery variable, measuring differences in GTMK effects between non-operative and operative subgroups. Complete regression results are provided in Table S11.
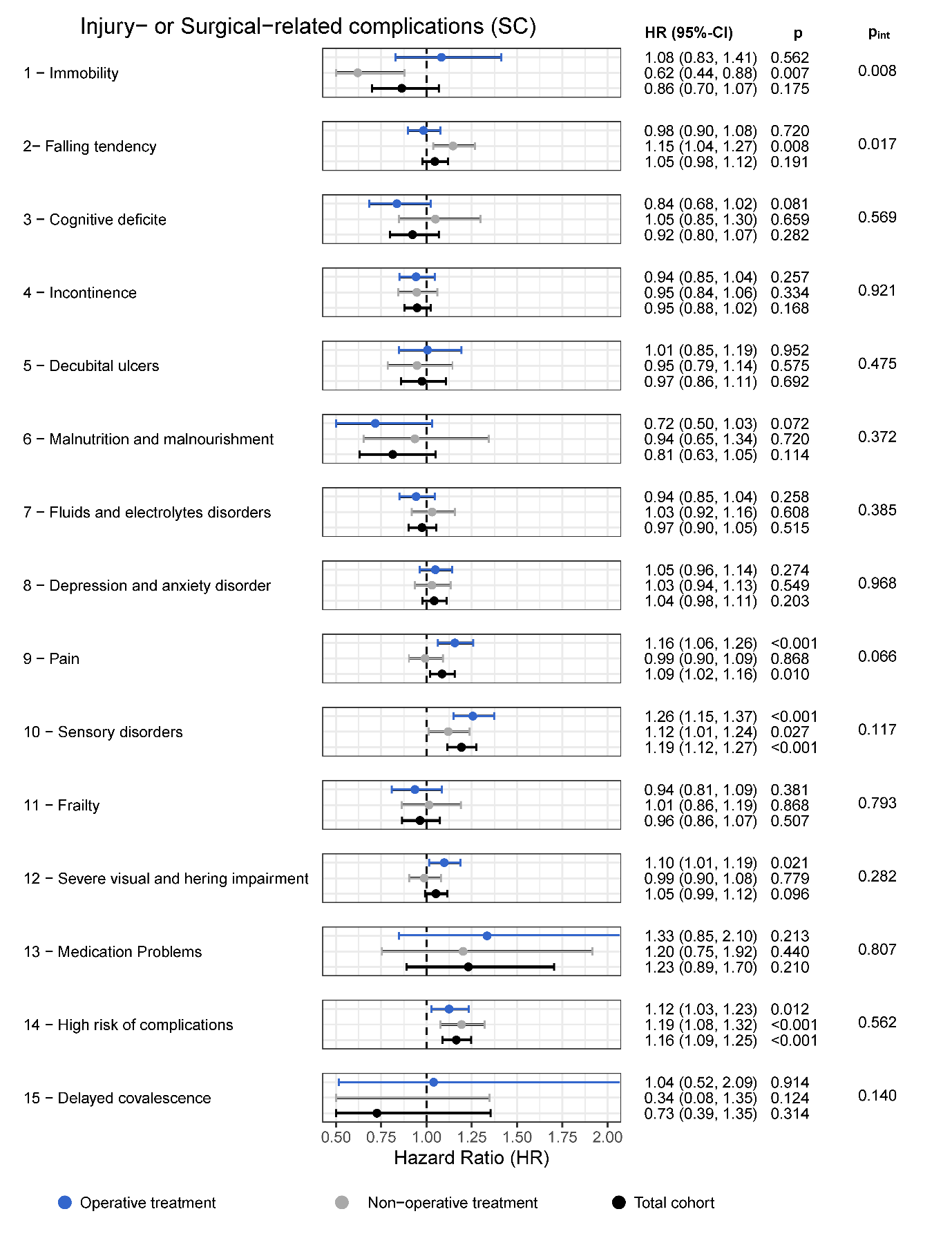


Figure S8: Influence of individual GTMK on Minor Outpatient Complications (MOC), adjusted for patient risk profile. The analysis was performed separately for non-operative patients (grey), operative patients (blue), and the overall cohort (black), including only patients who remained event-free after 3 months. Interaction p-values (p_int) were calculated using a Cox regression model with interaction terms between each GTMK and the binary surgery variable, measuring differences in GTMK effects between non-operative and operative subgroups. Complete regression results are provided in Table S12.


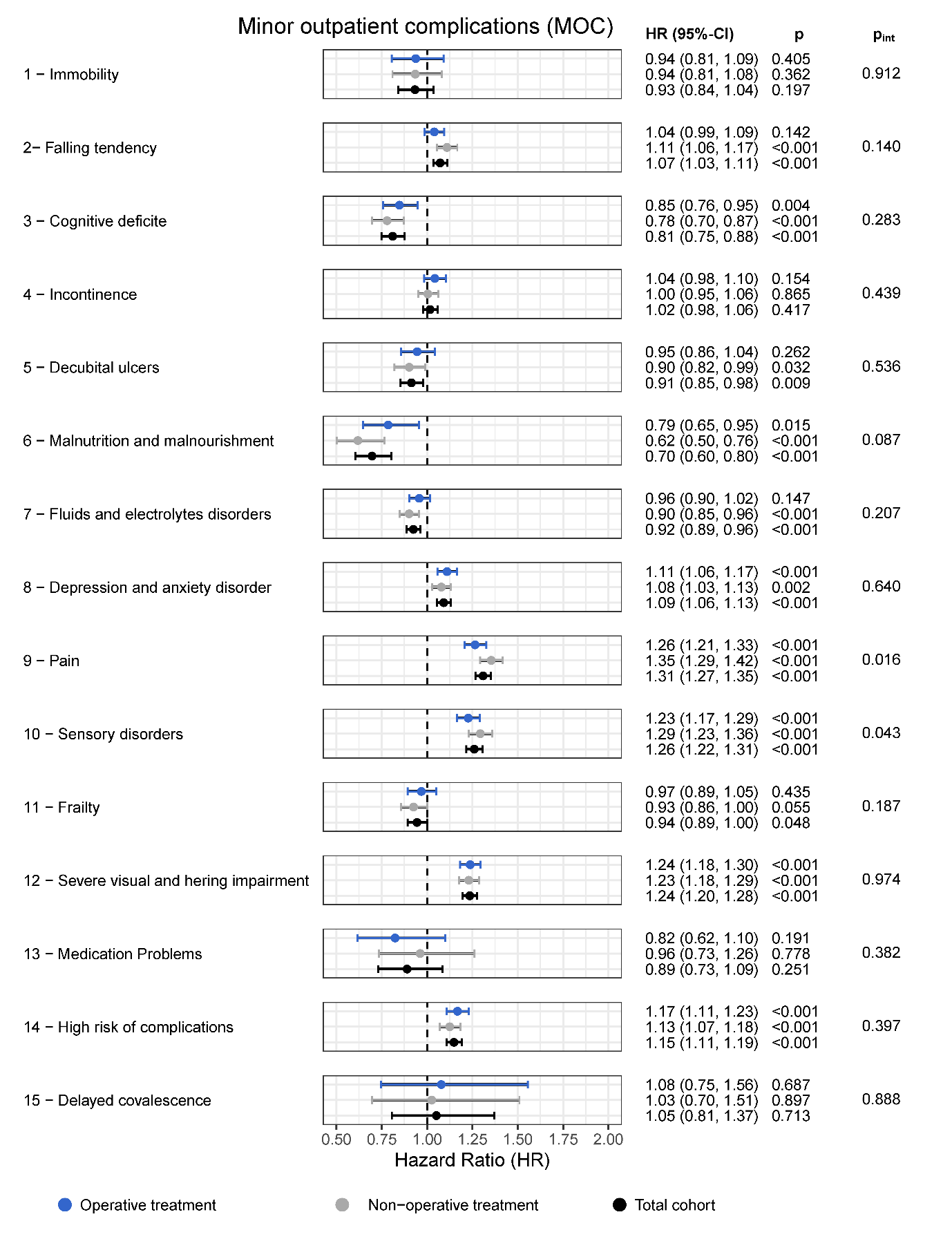


Table S3: Results of the Cox regression with mortality as the endpoint, including only patients who remained event-free after 3 months and the treatment group, number of present GTMK at PHF diagnosis, patient (risk) profile, events occurring within 21 days after PHF diagnosis, and an end of follow-up 3 months after the start of the observation.

| **Mortality** | | | |
| --- | --- | --- | --- |
| **Variable** | | **HR (95%-CI)** | **p** |
| Treatment group  (Reference group: non-operative treatment) | sLPF | 0.84 (0.82, 0.87) | <0.001 |
|  | LPF | 0.94 (0.89, 1.01) | 0.076 |
|  | RTSA | 0.87 (0.83, 0.90) | <0.001 |
|  | Others | 0.94 (0.91, 0.98) | <0.001 |
| Number of geriatric-typical characteristic complexes (GTMK) present at PHF diagnosis  (Reference group: no GTMK) | 1 GTMK | 0.90 (0.85, 0.96) | <0.001 |
|  | 2 GTMK | 0.92 (0.87, 0.97) | 0.003 |
|  | 3 GTMK | 0.99 (0.94, 1.05) | 0.716 |
|  | 4 GTMK | 1.07 (1.01, 1.13) | 0.025 |
|  | 5 GTMK | 1.14 (1.08, 1.21) | <0.001 |
|  | 6 GTMK | 1.21 (1.14, 1.29) | <0.001 |
|  | 7 GTMK | 1.29 (1.21, 1.38) | <0.001 |
|  | 8 GTMK | 1.33 (1.24, 1.44) | <0.001 |
|  | 9 and more GTMK | 1.49 (1.38, 1.62) | <0.001 |
| Age in years at PHF diagnosis | | 1.10 (1.10, 1.10) | <0.001 |
| Female Sex | | 0.62 (0.61, 0.64) | <0.001 |
| Comorbidities | Cancer | 1.05 (1.02, 1.07) | <0.001 |
|  | Diabetes | 1.23 (1.20, 1.26) | <0.001 |
|  | Dementia | 1.76 (1.71, 1.81) | <0.001 |
|  | Chronic polyarthritis | 1.03 (0.99, 1.08) | 0.159 |
|  | Obesity | 0.91 (0.89, 0.94) | <0.001 |
|  | Nicotine abuse | 1.56 (1.49, 1.63) | <0.001 |
|  | Parkinson | 1.41 (1.35, 1.48) | <0.001 |
|  | Rotator cuff rupture | 0.86 (0.80, 0.92) | <0.001 |
|  | Alcohol abuse | 1.67 (1.59, 1.75) | <0.001 |
|  | Previous stroke | 1.05 (1.02, 1.07) | <0.001 |
|  | Omarthrosis | 0.86 (0.80, 0.92) | <0.001 |
|  | Frozen shoulder | 0.79 (0.75, 0.84) | <0.001 |
|  | Atrial fibrillation and atrial flutter | 1.22 (1.19, 1.26) | <0.001 |
|  | Congestive heart failure | 1.26 (1.23, 1.29) | <0.001 |
|  | Coronary heart disease | 0.98 (0.95, 1.00) | 0.074 |
|  | Hypertonus | 1.02 (0.99, 1.06) | 0.184 |
|  | Atherosclerosis | 1.01 (0.98, 1.03) | 0.664 |
|  | Chronic kidney disease | 1.21 (1.18, 1.24) | <0.001 |
|  | Any anticoagulant | 1.14 (1.11, 1.17) | <0.001 |
|  | Vitamin D or Calcium | 0.98 (0.89, 1.07) | 0.648 |
|  | Bisphosphonate | 0.93 (0.85, 1.02) | 0.144 |
|  | Osteoporosis | 0.96 (0.94, 0.99) | 0.002 |
|  | Any osteoporosis medication | 1.18 (1.07, 1.31) | 0.001 |
| Outpatient sector | | 0.78 (0.76, 0.81) | <0.001 |
| Event within 21 days after diagnosis | Treatment change | 0.94 (0.87, 1.01) | 0.087 |
|  | Thromboembolic event | 1.07 (0.65, 1.74) | 0.794 |
|  | Injury-related event | 0.81 (0.75, 0.88) | <0.001 |
|  | Major adverse event | 1.67 (1.23, 2.26) | 0.001 |
|  | Minor outpatient event | 0.83 (0.77, 0.89) | <0.001 |

Table S4: Results of the Cox regression with major adverse events (MAE) as the endpoint, including only patients who remained event-free after 3 months and the treatment group, number of present GTMK at PHF diagnosis, patient (risk) profile, events occurring within 21 days after PHF diagnosis, and an end of follow-up 3 months after the start of the observation.

| **Major adverse events (MAE)** | | | |
| --- | --- | --- | --- |
| **Variable** | | **HR (95%-CI)** | **p** |
| Treatment group  (Reference group: non-operative treatment) | sLPF | 0.85 (0.82, 0.88) | <0.001 |
|  | LPF | 0.93 (0.87, 0.98) | 0.014 |
|  | RTSA | 0.87 (0.84, 0.90) | <0.001 |
|  | Others | 0.94 (0.91, 0.97) | <0.001 |
| Number of geriatric-typical characteristic complexes (GTMK) present at PHF diagnosis  (Reference group: no GTMK) | 1 GTMK | 0.94 (0.89, 0.99) | 0.025 |
|  | 2 GTMK | 0.94 (0.89, 0.99) | 0.026 |
|  | 3 GTMK | 1.03 (0.98, 1.08) | 0.312 |
|  | 4 GTMK | 1.10 (1.04, 1.16) | <0.001 |
|  | 5 GTMK | 1.18 (1.11, 1.24) | <0.001 |
|  | 6 GTMK | 1.24 (1.17, 1.31) | <0.001 |
|  | 7 GTMK | 1.32 (1.24, 1.41) | <0.001 |
|  | 8 GTMK | 1.38 (1.28, 1.48) | <0.001 |
|  | 9 and more GTMK | 1.50 (1.39, 1.62) | <0.001 |
| Age in years at PHF diagnosis | | 1.08 (1.08, 1.08) | <0.001 |
| Female Sex | | 0.66 (0.64, 0.68) | <0.001 |
| Comorbidities | Cancer | 1.05 (1.03, 1.07) | <0.001 |
|  | Diabetes | 1.21 (1.18, 1.23) | <0.001 |
|  | Dementia | 1.66 (1.62, 1.71) | <0.001 |
|  | Chronic polyarthritis | 1.06 (1.02, 1.11) | 0.004 |
|  | Obesity | 0.93 (0.90, 0.95) | <0.001 |
|  | Nicotine abuse | 1.45 (1.39, 1.52) | <0.001 |
|  | Parkinson | 1.36 (1.30, 1.42) | <0.001 |
|  | Rotator cuff rupture | 0.89 (0.83, 0.95) | 0.001 |
|  | Alcohol abuse | 1.60 (1.53, 1.67) | <0.001 |
|  | Previous stroke | 1.08 (1.05, 1.10) | <0.001 |
|  | Omarthrosis | 0.92 (0.86, 0.98) | 0.006 |
|  | Frozen shoulder | 0.83 (0.79, 0.87) | <0.001 |
|  | Atrial fibrillation and atrial flutter | 1.16 (1.13, 1.19) | <0.001 |
|  | Congestive heart failure | 1.23 (1.20, 1.26) | <0.001 |
|  | Coronary heart disease | 0.99 (0.96, 1.01) | 0.278 |
|  | Hypertonus | 1.05 (1.01, 1.08) | 0.004 |
|  | Atherosclerosis | 1.00 (0.98, 1.03) | 0.738 |
|  | Chronic kidney disease | 1.20 (1.18, 1.23) | <0.001 |
|  | Any anticoagulant | 1.14 (1.11, 1.17) | <0.001 |
|  | Vitamin D or Calcium | 0.99 (0.91, 1.09) | 0.88 |
|  | Bisphosphonate | 0.95 (0.87, 1.04) | 0.264 |
|  | Osteoporosis | 0.97 (0.95, 0.99) | 0.016 |
|  | Any osteoporosis medication | 1.15 (1.04, 1.26) | 0.007 |
| Outpatient sector | | 0.80 (0.78, 0.83) | <0.001 |
| Event within 21 days after diagnosis | Treatment change | 1.00 (0.93, 1.07) | 0.974 |
|  | Thromboembolic event | 1.05 (0.62, 1.79) | 0.847 |
|  | Injury-related event | 0.80 (0.75, 0.87) | <0.001 |
|  | Major adverse event | 1.61 (1.20, 2.16) | 0.001 |
|  | Minor outpatient event | 0.85 (0.80, 0.91) | <0.001 |

Table S5: Results of the Cox regression with thromboembolic event (TE) as the endpoint, including only patients who remained event-free after 3 months and the treatment group, number of present GTMK at PHF diagnosis, patient (risk) profile, events occurring within 21 days after PHF diagnosis, and an end of follow-up 3 months after the start of the observation.

| **Thromboembolic events (TE)** | | | |
| --- | --- | --- | --- |
| **Variable** | | **HR (95%-CI)** | **p** |
| Treatment group  (Reference group: non-operative treatment) | sLPF | 0.87 (0.84, 0.89) | <0.001 |
|  | LPF | 0.92 (0.87, 0.98) | 0.009 |
|  | RTSA | 0.90 (0.87, 0.93) | <0.001 |
|  | Others | 0.95 (0.92, 0.98) | 0.003 |
| Number of geriatric-typical characteristic complexes (GTMK) present at PHF diagnosis  (Reference group: no GTMK) | 1 GTMK | 0.95 (0.90, 1.00) | 0.036 |
|  | 2 GTMK | 0.96 (0.91, 1.00) | 0.075 |
|  | 3 GTMK | 1.03 (0.98, 1.08) | 0.250 |
|  | 4 GTMK | 1.11 (1.05, 1.17) | <0.001 |
|  | 5 GTMK | 1.18 (1.12, 1.25) | <0.001 |
|  | 6 GTMK | 1.26 (1.19, 1.33) | <0.001 |
|  | 7 GTMK | 1.34 (1.26, 1.42) | <0.001 |
|  | 8 GTMK | 1.36 (1.27, 1.45) | <0.001 |
|  | 9 and more GTMK | 1.46 (1.36, 1.58) | <0.001 |
| Age in years at PHF diagnosis | | 1.07 (1.07, 1.08) | <0.001 |
| Female Sex | | 0.65 (0.63, 0.67) | <0.001 |
| Comorbidities | Cancer | 1.03 (1.01, 1.05) | 0.004 |
|  | Diabetes | 1.24 (1.21, 1.26) | <0.001 |
|  | Dementia | 1.59 (1.55, 1.64) | <0.001 |
|  | Chronic polyarthritis | 1.07 (1.03, 1.11) | <0.001 |
|  | Obesity | 0.97 (0.94, 0.99) | 0.009 |
|  | Nicotine abuse | 1.45 (1.40, 1.51) | <0.001 |
|  | Parkinson | 1.35 (1.30, 1.41) | <0.001 |
|  | Rotator cuff rupture | 0.92 (0.86, 0.98) | 0.010 |
|  | Alcohol abuse | 1.63 (1.56, 1.70) | <0.001 |
|  | Previous stroke | 1.08 (1.06, 1.11) | <0.001 |
|  | Omarthrosis | 0.92 (0.87, 0.98) | 0.007 |
|  | Frozen shoulder | 0.85 (0.81, 0.89) | <0.001 |
|  | Atrial fibrillation and atrial flutter | 1.17 (1.14, 1.20) | <0.001 |
|  | Congestive heart failure | 1.22 (1.19, 1.25) | <0.001 |
|  | Coronary heart disease | 1.02 (0.99, 1.04) | 0.137 |
|  | Hypertonus | 1.10 (1.07, 1.14) | <0.001 |
|  | Atherosclerosis | 1.01 (0.98, 1.04) | 0.482 |
|  | Chronic kidney disease | 1.25 (1.22, 1.28) | <0.001 |
|  | Any anticoagulant | 1.16 (1.13, 1.19) | <0.001 |
|  | Vitamin D or Calcium | 1.00 (0.92, 1.09) | 0.981 |
|  | Bisphosphonate | 0.96 (0.88, 1.05) | 0.335 |
|  | Osteoporosis | 0.97 (0.95, 0.99) | 0.007 |
|  | Any osteoporosis medication | 1.13 (1.03, 1.24) | 0.013 |
| Outpatient sector | | 0.82 (0.80, 0.84) | <0.001 |
| Event within 21 days after diagnosis | Treatment change | 1.00 (0.94, 1.07) | 0.918 |
|  | Thromboembolic event | 0.93 (0.52, 1.65) | 0.804 |
|  | Injury-related event | 0.85 (0.79, 0.91) | <0.001 |
|  | Major adverse event | 1.73 (1.22, 2.45) | 0.002 |
|  | Minor outpatient event | 0.86 (0.81, 0.91) | <0.001 |

Table S6: Results of the Cox regression with injury- or operative related complications (SC) as the endpoint and death as competing event, including only patients who remained event-free after 3 months and the treatment group, number of present GTMK at PHF diagnosis, patient (risk) profile, events occurring within 21 days after PHF diagnosis, and an end of follow-up 3 months after the start of the observation.

| **Injury- or operative related complications (SC)** | | | |
| --- | --- | --- | --- |
| Variable | | HR (95%-CI) | p |
| Treatment group  (Reference group: non-operative treatment) | sLPF | 1.07 (0.97, 1.18) | 0.190 |
|  | LPF | 0.82 (0.67, 1.01) | 0.056 |
|  | RTSA | 1.39 (1.25, 1.55) | <0.001 |
|  | Others | 0.97 (0.87, 1.08) | 0.563 |
| Number of geriatric-typical characteristic complexes (GTMK) present at PHF diagnosis  (Reference group: no GTMK) | 1 GTMK | 1.08 (0.94, 1.24) | 0.295 |
|  | 2 GTMK | 1.14 (1.00, 1.30) | 0.054 |
|  | 3 GTMK | 1.21 (1.06, 1.39) | 0.005 |
|  | 4 GTMK | 1.26 (1.10, 1.45) | 0.001 |
|  | 5 GTMK | 1.33 (1.14, 1.54) | <0.001 |
|  | 6 GTMK | 1.39 (1.18, 1.64) | <0.001 |
|  | 7 GTMK | 1.43 (1.18, 1.73) | <0.001 |
|  | 8 GTMK | 1.35 (1.06, 1.72) | 0.016 |
|  | 9 and more GTMK | 1.31 (0.98, 1.77) | 0.073 |
| Age in years at PHF diagnosis | | 0.96 (0.95, 0.96) | <0.001 |
| Female Sex | | 0.97 (0.89, 1.06) | 0.506 |
| Comorbidities | Cancer | 1.03 (0.96, 1.10) | 0.371 |
|  | Diabetes | 1.01 (0.94, 1.08) | 0.769 |
|  | Dementia | 0.67 (0.59, 0.76) | <0.001 |
|  | Chronic polyarthritis | 1.13 (1.02, 1.26) | 0.021 |
|  | Obesity | 1.18 (1.10, 1.26) | <0.001 |
|  | Nicotine abuse | 0.96 (0.85, 1.07) | 0.443 |
|  | Parkinson | 1.12 (0.95, 1.31) | 0.181 |
|  | Rotator cuff rupture | 1.17 (1.00, 1.37) | 0.050 |
|  | Alcohol abuse | 1.17 (1.03, 1.31) | 0.012 |
|  | Previous stroke | 0.97 (0.90, 1.04) | 0.341 |
|  | Omarthrosis | 1.13 (0.97, 1.32) | 0.128 |
|  | Frozen shoulder | 1.23 (1.09, 1.39) | <0.001 |
|  | Atrial fibrillation and atrial flutter | 1.00 (0.91, 1.10) | 0.966 |
|  | Congestive heart failure | 0.95 (0.87, 1.03) | 0.230 |
|  | Coronary heart disease | 1.04 (0.96, 1.12) | 0.328 |
|  | Hypertonus | 1.02 (0.94, 1.11) | 0.603 |
|  | Atherosclerosis | 1.05 (0.96, 1.14) | 0.268 |
|  | Chronic kidney disease | 0.95 (0.87, 1.03) | 0.188 |
|  | Any anticoagulant | 1.01 (0.94, 1.09) | 0.770 |
|  | Vitamin D or Calcium | 0.99 (0.78, 1.27) | 0.965 |
|  | Bisphosphonate | 0.99 (0.77, 1.28) | 0.963 |
|  | Osteoporosis | 1.11 (1.04, 1.19) | 0.002 |
|  | Any osteoporosis medication | 1.11 (0.84, 1.47) | 0.455 |
| Outpatient sector | | 0.85 (0.78, 0.93) | <0.001 |
| Event within 21 days after diagnosis | Treatment change | 19.27 (17.45, 21.28) | <0.001 |
|  | Thromboembolic event | 1.92 (0.15, 24.72) | 0.617 |
|  | Injury-related event | 1.45 (1.23, 1.72) | <0.001 |
|  | Major adverse event | 0.52 (0.09, 3.18) | 0.479 |
|  | Minor outpatient event | 1.15 (1, 1.34) | 0.057 |

Table S7: Results of the Cox regression with minor outpatient complications (SC) as the endpoint and death as competing event, including only patients who remained event-free after 3 months and the treatment group, number of present GTMK at PHF diagnosis, patient (risk) profile, events occurring within 21 days after PHF diagnosis, and an end of follow-up 3 months after the start of the observation.

| **Minor outpatient complications (MOC)** | | | |
| --- | --- | --- | --- |
| Variable | | HR (95%-CI) | p |
| Treatment group  (Reference group: non-operative treatment) | sLPF | 0.85 (0.82, 0.88) | <0.001 |
|  | LPF | 0.93 (0.87, 0.98) | 0.014 |
|  | RTSA | 0.87 (0.84, 0.9) | <0.001 |
|  | Others | 0.94 (0.91, 0.97) | <0.001 |
| Number of geriatric-typical characteristic complexes (GTMK) present at PHF diagnosis  (Reference group: no GTMK) | 1 GTMK | 0.94 (0.89, 0.99) | 0.025 |
|  | 2 GTMK | 0.94 (0.89, 0.99) | 0.026 |
|  | 3 GTMK | 1.03 (0.98, 1.08) | 0.312 |
|  | 4 GTMK | 1.10 (1.04, 1.16) | <0.001 |
|  | 5 GTMK | 1.18 (1.11, 1.24) | <0.001 |
|  | 6 GTMK | 1.24 (1.17, 1.31) | <0.001 |
|  | 7 GTMK | 1.32 (1.24, 1.41) | <0.001 |
|  | 8 GTMK | 1.38 (1.28, 1.48) | <0.001 |
|  | 9 and more GTMK | 1.50 (1.39, 1.62) | <0.001 |
| Age in years at PHF diagnosis | | 1.08 (1.08, 1.08) | <0.001 |
| Female Sex | | 0.66 (0.64, 0.68) | <0.001 |
| Comorbidities | Cancer | 1.05 (1.03, 1.07) | <0.001 |
|  | Diabetes | 1.21 (1.18, 1.23) | <0.001 |
|  | Dementia | 1.66 (1.62, 1.71) | <0.001 |
|  | Chronic polyarthritis | 1.06 (1.02, 1.11) | 0.004 |
|  | Obesity | 0.93 (0.90, 0.95) | <0.001 |
|  | Nicotine abuse | 1.45 (1.39, 1.52) | <0.001 |
|  | Parkinson | 1.36 (1.30, 1.42) | <0.001 |
|  | Rotator cuff rupture | 0.89 (0.83, 0.95) | 0.001 |
|  | Alcohol abuse | 1.60 (1.53, 1.67) | <0.001 |
|  | Previous stroke | 1.08 (1.05, 1.10) | <0.001 |
|  | Omarthrosis | 0.92 (0.86, 0.98) | 0.006 |
|  | Frozen shoulder | 0.83 (0.79, 0.87) | <0.001 |
|  | Atrial fibrillation and atrial flutter | 1.16 (1.13, 1.19) | <0.001 |
|  | Congestive heart failure | 1.23 (1.20, 1.26) | <0.001 |
|  | Coronary heart disease | 0.99 (0.96, 1.01) | 0.278 |
|  | Hypertonus | 1.05 (1.01, 1.08) | 0.004 |
|  | Atherosclerosis | 1.00 (0.98, 1.03) | 0.738 |
|  | Chronic kidney disease | 1.20 (1.18, 1.23) | <0.001 |
|  | Any anticoagulant | 1.14 (1.11, 1.17) | <0.001 |
|  | Vitamin D or Calcium | 0.99 (0.91, 1.09) | 0.88 |
|  | Bisphosphonate | 0.95 (0.87, 1.04) | 0.264 |
|  | Osteoporosis | 0.97 (0.95, 0.99) | 0.016 |
|  | Any osteoporosis medication | 1.15 (1.04, 1.26) | 0.007 |
| Outpatient sector | | 0.80 (0.78, 0.83) | <0.001 |
| Event within 21 days after diagnosis | Treatment change | 1.00 (0.93, 1.07) | 0.974 |
|  | Thromboembolic event | 1.05 (0.62, 1.79) | 0.847 |
|  | Injury-related event | 0.80 (0.75, 0.87) | <0.001 |
|  | Major adverse event | 1.61 (1.20, 2.16) | 0.001 |
|  | Minor outpatient event | 0.85 (0.80, 0.91) | <0.001 |

Table S8: Results of three Cox regression models with mortality as an outcome for the overall cohort, the subgroup of those who underwent surgery within 21 days of PHF diagnosis, and those who did not, including only patients who remained event-free after 3 months. Included variables are current GTMK at the time of PHF diagnosis, patient (risk) profile, and events that occurred within 21 days of PHF diagnosis, with an end of follow-up 3 months after the start of observation. The difference between operative and non-operative treatment within individual GTMK is quantified with the interaction p value p_int. This comes from a Cox regression which, in addition to the influencing factors listed here, also includes the interaction between operation and the individual GTMK.

| **Mortality** | | | | | | | |
| --- | --- | --- | --- | --- | --- | --- | --- |
|  | **Total cohort** | | **Subgroup:  Operative Treatment** | | **Subgroup:  Non-Operative Treatment** | | **OP vs no OP** |
| **Variable** | **HR (95%-CI)** | **p** | **HR (95%-CI)** | **P** | **HR (95%-CI)** | **p** | **P_int_** |
| Immobility | 1.12 (1.06, 1.18) | <0.001 | 1.11 (1.02, 1.21) | 0.013 | 1.12 (1.05, 1.20) | <0.001 | 0.813 |
| Falling tendency | 1.05 (1.02, 1.07) | <0.001 | 1.08 (1.04, 1.12) | <0.001 | 1.02 (0.99, 1.05) | 0.166 | 0.056 |
| Cognitive deficits | 1.25 (1.20, 1.30) | <0.001 | 1.24 (1.17, 1.32) | <0.001 | 1.25 (1.19, 1.31) | <0.001 | 0.591 |
| Incontinence | 1.15 (1.12, 1.18) | <0.001 | 1.13 (1.09, 1.17) | <0.001 | 1.16 (1.12, 1.20) | <0.001 | 0.245 |
| Decubital ulcers | 1.38 (1.34, 1.43) | <0.001 | 1.43 (1.35, 1.50) | <0.001 | 1.35 (1.30, 1.41) | <0.001 | 0.111 |
| Malnutrition and malnourishment | 1.43 (1.36, 1.51) | <0.001 | 1.53 (1.41, 1.67) | <0.001 | 1.37 (1.28, 1.47) | <0.001 | 0.086 |
| Fluids and electrolytes disorders | 1.27 (1.24, 1.31) | <0.001 | 1.25 (1.20, 1.30) | <0.001 | 1.29 (1.25, 1.33) | <0.001 | 0.232 |
| Depression and anxiety disorders | 1.07 (1.04, 1.09) | <0.001 | 1.07 (1.03, 1.11) | <0.001 | 1.07 (1.03, 1.10) | <0.001 | 0.999 |
| Pain | 0.90 (0.88, 0.92) | <0.001 | 0.87 (0.84, 0.91) | <0.001 | 0.91 (0.89, 0.94) | <0.001 | 0.064 |
| Sensory disorders | 0.96 (0.94, 0.99) | 0.003 | 1.00 (0.96, 1.04) | 0.973 | 0.94 (0.91, 0.97) | <0.001 | 0.005 |
| Frailty | 1.03 (1.00, 1.07) | 0.04 | 1.03 (0.98, 1.08) | 0.325 | 1.04 (1.00, 1.08) | 0.072 | 0.615 |
| Severe visual and hearing impairment | 0.84 (0.82, 0.86) | <0.001 | 0.83 (0.81, 0.86) | <0.001 | 0.85 (0.82, 0.87) | <0.001 | 0.652 |
| Medication problems | 0.95 (0.86, 1.05) | 0.305 | 0.89 (0.76, 1.04) | 0.157 | 0.99 (0.87, 1.13) | 0.913 | 0.379 |
| High risk of complications | 1.02 (0.99, 1.04) | 0.218 | 0.96 (0.93, 1.00) | 0.067 | 1.06 (1.02, 1.10) | <0.001 | <0.001 |
| Delayed convalescence | 0.89 (0.77, 1.03) | 0.115 | 0.83 (0.66, 1.03) | 0.09 | 0.94 (0.78, 1.15) | 0.563 | 0.371 |
| Age in years at PHF diagnosis | 1.10 (1.10, 1.10) | <0.001 | 1.10 (1.09, 1.10) | <0.001 | 1.10 (1.09, 1.10) | <0.001 |  |
| Female Sex | 0.61 (0.60, 0.63) | <0.001 | 0.61 (0.58, 0.64) | <0.001 | 0.62 (0.60, 0.64) | <0.001 |  |
| Cancer | 1.06 (1.03, 1.08) | <0.001 | 1.06 (1.02, 1.10) | 0.002 | 1.06 (1.03, 1.09) | <0.001 |  |
| Diabetes | 1.25 (1.22, 1.28) | <0.001 | 1.26 (1.21, 1.30) | <0.001 | 1.24 (1.20, 1.28) | <0.001 |  |
| Dementia | 1.46 (1.41, 1.52) | <0.001 | 1.50 (1.41, 1.59) | <0.001 | 1.42 (1.35, 1.49) | <0.001 |  |
| Chronic polyarthritis | 1.06 (1.01, 1.10) | 0.012 | 1.06 (0.99, 1.13) | 0.101 | 1.05 (1.00, 1.11) | 0.067 |  |
| Obesity | 0.93 (0.91, 0.96) | <0.001 | 0.93 (0.89, 0.97) | <0.001 | 0.94 (0.91, 0.98) | 0.002 |  |
| Nicotine abuse | 1.53 (1.47, 1.60) | <0.001 | 1.52 (1.42, 1.63) | <0.001 | 1.54 (1.45, 1.64) | <0.001 |  |
| Parkinson | 1.37 (1.31, 1.43) | <0.001 | 1.34 (1.26, 1.44) | <0.001 | 1.39 (1.31, 1.47) | <0.001 |  |
| Rotator cuff rupture | 0.85 (0.79, 0.92) | <0.001 | 0.93 (0.85, 1.02) | 0.101 | 0.79 (0.69, 0.89) | <0.001 |  |
| Alcohol abuse | 1.58 (1.50, 1.65) | <0.001 | 1.58 (1.47, 1.69) | <0.001 | 1.58 (1.48, 1.68) | <0.001 |  |
| Previous stroke | 1.06 (1.03, 1.08) | <0.001 | 1.04 (1, 1.08) | 0.039 | 1.07 (1.04, 1.10) | <0.001 |  |
| Omarthrosis | 0.9 (0.85, 0.97) | 0.004 | 0.95 (0.85, 1.06) | 0.345 | 0.88 (0.81, 0.96) | 0.003 |  |
| Frozen shoulder | 0.82 (0.78, 0.87) | <0.001 | 0.82 (0.75, 0.90) | <0.001 | 0.83 (0.77, 0.89) | <0.001 |  |
| Atrial fibrillation and atrial flutter | 1.22 (1.19, 1.26) | <0.001 | 1.26 (1.20, 1.32) | <0.001 | 1.20 (1.15, 1.24) | <0.001 |  |
| Congestive heart failure | 1.20 (1.17, 1.24) | <0.001 | 1.2 (1.15, 1.25) | <0.001 | 1.21 (1.17, 1.25) | <0.001 |  |
| Coronary heart disease | 1.00 (0.97, 1.02) | 0.858 | 1.00 (0.96, 1.04) | 0.925 | 1.00 (0.97, 1.03) | 0.863 |  |
| Hypertonus | 1.02 (0.99, 1.06) | 0.178 | 1.06 (1.01, 1.12) | 0.029 | 1.00 (0.96, 1.04) | 0.982 |  |
| Atherosclerosis | 1.02 (1.00, 1.05) | 0.107 | 1.04 (1.00, 1.09) | 0.063 | 1.01 (0.97, 1.05) | 0.564 |  |
| Chronic kidney disease | 1.18 (1.15, 1.21) | <0.001 | 1.20 (1.16, 1.25) | <0.001 | 1.16 (1.12, 1.2) | <0.001 |  |
| Any anticoagulant | 1.12 (1.09, 1.14) | <0.001 | 1.09 (1.05, 1.14) | <0.001 | 1.13 (1.09, 1.16) | <0.001 |  |
| Vitamin D or Calcium | 0.98 (0.89, 1.07) | 0.62 | 0.97 (0.84, 1.12) | 0.68 | 0.98 (0.86, 1.11) | 0.725 |  |
| Bisphosphonate | 0.94 (0.86, 1.03) | 0.209 | 0.99 (0.85, 1.15) | 0.867 | 0.91 (0.81, 1.03) | 0.14 |  |
| Osteoporosis | 0.98 (0.95, 1.00) | 0.072 | 0.97 (0.93, 1.01) | 0.099 | 0.99 (0.95, 1.02) | 0.388 |  |
| Any osteoporosis medication | 1.20 (1.08, 1.33) | <0.001 | 1.18 (1, 1.39) | 0.049 | 1.21 (1.05, 1.38) | 0.007 |  |
| Outpatient sector | 0.85 (0.83, 0.87) | <0.001 | 1.50 (1.11, 2.02) | 0.008 | 0.79 (0.77, 0.81) | <0.001 |  |
| Treatment change within 21 days after diagnosis | 0.91 (0.84, 0.97) | 0.007 | 0.94 (0.88, 1.02) | 0.122 |  |  |  |
| Thromboembolic event within 21 days after diagnosis | 1.19 (0.73, 1.93) | 0.482 | 1.40 (0.54, 3.65) | 0.488 | 1.09 (0.62, 1.89) | 0.772 |  |
| Injury-related event within 21 days after diagnosis | 0.85 (0.78, 0.92) | <0.001 | 0.87 (0.76, 1.00) | 0.053 | 0.80 (0.73, 0.89) | <0.001 |  |
| Major adverse event within 21 days after diagnosis | 1.66 (1.23, 2.25) | <0.001 | 1.53 (0.78, 3.02) | 0.216 | 1.67 (1.19, 2.34) | 0.003 |  |
| Minor outpatient event within 21 days after diagnosis | 0.84 (0.79, 0.90) | <0.001 | 0.85 (0.72, 10) | 0.05 | 0.86 (0.80, 0.93) | <0.001 |  |

Table S9: Results of three Cox regression models with major adverse events (MAE) as an outcome for the overall cohort, the subgroup of those who underwent surgery within 21 days of PHF diagnosis, and those who did not, including only patients who remained event-free after 3 months. Included variables are current GTMK at the time of PHF diagnosis, patient (risk) profile, and events that occurred within 21 days of PHF diagnosis, with an end of follow-up 3 months after the start of observation. The difference between operative and non-operative treatment within individual GTMK is quantified with the interaction p value p_int. This comes from a Cox regression which, in addition to the influencing factors listed here, also includes the interaction between operation and the individual GTMK.

| **Major Adverse Events (MAE)** | | | | | | | |
| --- | --- | --- | --- | --- | --- | --- | --- |
|  | **Total cohort** | | **Subgroup:  Operative Treatment** | | **Subgroup:  Non-Operative Treatment** | | **OP vs no OP** |
| **Variable** | **HR (95%-CI)** | **p** | **HR (95%-CI)** | **P** | **HR (95%-CI)** | **p** | **P_int_** |
| Immobility | 1.09 (1.04, 1.14) | <0.001 | 1.10 (1.01, 1.19) | 0.022 | 1.09 (1.02, 1.15) | 0.01 | 0.846 |
| Falling tendency | 1.06 (1.04, 1.08) | <0.001 | 1.07 (1.03, 1.11) | <0.001 | 1.05 (1.02, 1.08) | 0.002 | 0.451 |
| Cognitive deficits | 1.18 (1.14, 1.22) | <0.001 | 1.19 (1.12, 1.26) | <0.001 | 1.17 (1.12, 1.23) | <0.001 | 0.720 |
| Incontinence | 1.12 (1.10, 1.15) | <0.001 | 1.12 (1.08, 1.16) | <0.001 | 1.12 (1.09, 1.15) | <0.001 | 0.758 |
| Decubital ulcers | 1.31 (1.27, 1.36) | <0.001 | 1.37 (1.30, 1.44) | <0.001 | 1.28 (1.23, 1.33) | <0.001 | 0.043 |
| Malnutrition and malnourishment | 1.38 (1.31, 1.45) | <0.001 | 1.41 (1.30, 1.54) | <0.001 | 1.36 (1.27, 1.45) | <0.001 | 0.493 |
| Fluids and electrolytes disorders | 1.23 (1.20, 1.26) | <0.001 | 1.20 (1.16, 1.24) | <0.001 | 1.25 (1.21, 1.28) | <0.001 | 0.190 |
| Depression and anxiety disorders | 1.07 (1.05, 1.09) | <0.001 | 1.09 (1.06, 1.13) | <0.001 | 1.06 (1.03, 1.09) | <0.001 | 0.129 |
| Pain | 0.93 (0.91, 0.95) | <0.001 | 0.9 (0.87, 0.93) | <0.001 | 0.95 (0.92, 0.97) | <0.001 | 0.033 |
| Sensory disorders | 1.00 (0.98, 1.03) | 0.664 | 1.05 (1.01, 1.09) | 0.008 | 0.97 (0.95, 1.00) | 0.093 | <0.001 |
| Frailty | 1.00 (0.97, 1.03) | 0.948 | 0.98 (0.94, 1.03) | 0.532 | 1.01 (0.97, 1.05) | 0.677 | 0.345 |
| Severe visual and hearing impairment | 0.87 (0.85, 0.89) | <0.001 | 0.86 (0.83, 0.88) | <0.001 | 0.88 (0.86, 0.9) | <0.001 | 0.185 |
| Medication problems | 0.98 (0.89, 1.08) | 0.649 | 0.92 (0.79, 1.07) | 0.259 | 1.03 (0.91, 1.17) | 0.666 | 0.313 |
| High risk of complications | 1.03 (1.01, 1.06) | 0.008 | 1.00 (0.96, 1.03) | 0.871 | 1.06 (1.03, 1.10) | <0.001 | 0.021 |
| Delayed convalescence | 0.86 (0.75, 0.99) | 0.041 | 0.82 (0.66, 1.01) | 0.06 | 0.91 (0.75, 1.10) | 0.309 | 0.470 |
| Age in years at PHF diagnosis | 1.07 (1.07, 1.08) | <0.001 | 1.07 (1.07, 1.07) | <0.001 | 1.07 (1.07, 1.08) | <0.001 |  |
| Female Sex | 0.64 (0.62, 0.66) | <0.001 | 0.64 (0.61, 0.66) | <0.001 | 0.65 (0.63, 0.67) | <0.001 |  |
| Cancer | 1.04 (1.02, 1.06) | <0.001 | 1.04 (1.01, 1.08) | 0.011 | 1.04 (1.01, 1.07) | 0.006 |  |
| Diabetes | 1.24 (1.22, 1.27) | <0.001 | 1.25 (1.21, 1.29) | <0.001 | 1.24 (1.21, 1.28) | <0.001 |  |
| Dementia | 1.39 (1.34, 1.44) | <0.001 | 1.40 (1.32, 1.48) | <0.001 | 1.37 (1.31, 1.44) | <0.001 |  |
| Chronic polyarthritis | 1.09 (1.05, 1.13) | <0.001 | 1.10 (1.03, 1.16) | 0.003 | 1.08 (1.03, 1.14) | 0.002 |  |
| Obesity | 0.98 (0.95, 1.00) | 0.086 | 0.97 (0.94, 1.01) | 0.167 | 0.98 (0.95, 1.02) | 0.334 |  |
| Nicotine abuse | 1.43 (1.37, 1.49) | <0.001 | 1.43 (1.35, 1.52) | <0.001 | 1.42 (1.35, 1.51) | <0.001 |  |
| Parkinson | 1.31 (1.26, 1.37) | <0.001 | 1.27 (1.18, 1.35) | <0.001 | 1.34 (1.27, 1.42) | <0.001 |  |
| Rotator cuff rupture | 0.92 (0.86, 0.98) | 0.008 | 1.00 (0.92, 1.09) | 0.923 | 0.82 (0.74, 0.92) | <0.001 |  |
| Alcohol abuse | 1.56 (1.49, 1.63) | <0.001 | 1.59 (1.50, 1.70) | <0.001 | 1.53 (1.44, 1.62) | <0.001 |  |
| Previous stroke | 1.09 (1.06, 1.11) | <0.001 | 1.08 (1.04, 1.12) | <0.001 | 1.09 (1.06, 1.12) | <0.001 |  |
| Omarthrosis | 0.96 (0.90, 1.02) | 0.17 | 1.00 (0.90, 1.11) | 0.978 | 0.94 (0.87, 1.01) | 0.082 |  |
| Frozen shoulder | 0.87 (0.83, 0.92) | <0.001 | 0.87 (0.81, 0.95) | <0.001 | 0.87 (0.82, 0.93) | <0.001 |  |
| Atrial fibrillation and atrial flutter | 1.17 (1.14, 1.20) | <0.001 | 1.20 (1.15, 1.25) | <0.001 | 1.15 (1.11, 1.19) | <0.001 |  |
| Congestive heart failure | 1.18 (1.16, 1.21) | <0.001 | 1.20 (1.15, 1.24) | <0.001 | 1.17 (1.14, 1.21) | <0.001 |  |
| Coronary heart disease | 1.03 (1.01, 1.06) | 0.006 | 1.02 (0.98, 1.05) | 0.385 | 1.04 (1.01, 1.08) | 0.004 |  |
| Hypertonus | 1.11 (1.07, 1.14) | <0.001 | 1.14 (1.09, 1.19) | <0.001 | 1.09 (1.04, 1.13) | <0.001 |  |
| Atherosclerosis | 1.02 (1.00, 1.05) | 0.067 | 1.01 (0.97, 1.06) | 0.49 | 1.03 (1.00, 1.07) | 0.075 |  |
| Chronic kidney disease | 1.22 (1.20, 1.25) | <0.001 | 1.27 (1.22, 1.31) | <0.001 | 1.19 (1.16, 1.23) | <0.001 |  |
| Any anticoagulant | 1.14 (1.11, 1.16) | <0.001 | 1.12 (1.08, 1.16) | <0.001 | 1.15 (1.11, 1.18) | <0.001 |  |
| Vitamin D or Calcium | 1.00 (0.92, 1.09) | 0.996 | 1.03 (0.90, 1.18) | 0.652 | 0.98 (0.87, 1.10) | 0.711 |  |
| Bisphosphonate | 0.97 (0.89, 1.06) | 0.47 | 1.05 (0.91, 1.20) | 0.509 | 0.92 (0.82, 1.03) | 0.142 |  |
| Osteoporosis | 0.98 (0.96, 1.00) | 0.1 | 0.97 (0.94, 1.01) | 0.147 | 0.99 (0.96, 1.02) | 0.402 |  |
| Any osteoporosis medication | 1.13 (1.03, 1.24) | 0.012 | 1.08 (0.93, 1.25) | 0.295 | 1.16 (1.03, 1.32) | 0.019 |  |
| Outpatient sector | 0.87 (0.86, 0.89) | <0.001 | 1.30 (0.98, 1.73) | 0.07 | 0.82 (0.80, 0.85) | <0.001 |  |
| Treatment change within 21 days after diagnosis | 0.99 (0.92, 1.05) | 0.659 | 1.02 (0.95, 1.08) | 0.624 |  |  |  |
| Thromboembolic event within 21 days after diagnosis | 0.99 (0.56, 1.76) | 0.984 | 2.50 (0.82, 7.57) | 0.105 | 0.80 (0.42, 1.54) | 0.513 |  |
| Injury-related event within 21 days after diagnosis | 0.89 (0.83, 0.95) | <0.001 | 0.91 (0.80, 1.03) | 0.119 | 0.85 (0.78, 0.93) | <0.001 |  |
| Major adverse event within 21 days after diagnosis | 1.75 (1.24, 2.48) | 0.001 | 1.70 (0.84, 3.43) | 0.137 | 1.75 (1.19, 2.59) | 0.005 |  |
| Minor outpatient event within 21 days after diagnosis | 0.87 (0.82, 0.93) | <0.001 | 1.06 (0.79. 1.43) | 0.679 | 0.89 (0.83, 0.95) | 0.001 |  |

Table S10: Results of three Cox regression models with thromboembolic events (TE) as an outcome for the overall cohort, the subgroup of those who underwent surgery within 21 days of PHF diagnosis, and those who did not, including only patients who remained event-free after 3 months. Included variables are current GTMK at the time of PHF diagnosis, patient (risk) profile, and events that occurred within 21 days of PHF diagnosis, with an end of follow-up 3 months after the start of observation. The difference between operative and non-operative treatment within individual GTMK is quantified with the interaction p value p_int. This comes from a Cox regression which, in addition to the influencing factors listed here, also includes the interaction between operation and the individual GTMK.

| **Thromboembolic Events (TE)** | | | | | | | |
| --- | --- | --- | --- | --- | --- | --- | --- |
|  | **Total cohort** | | **Subgroup:  Operative Treatment** | | **Subgroup:  Non-Operative Treatment** | | **OP vs no OP** |
| **Variable** | **HR (95%-CI)** | **p** | **HR (95%-CI)** | **P** | **HR (95%-CI)** | **p** | **P_interaction_** |
| Immobility | 1.13 (1.07, 1.18) | <0.001 | 1.10 (1.01, 1.19) | 0.025 | 1.14 (1.07, 1.22) | <0.001 | 0.411 |
| Falling tendency | 1.06 (1.04, 1.09) | <0.001 | 1.09 (1.06, 1.13) | <0.001 | 1.04 (1.01, 1.07) | 0.017 | 0.038 |
| Cognitive deficits | 1.21 (1.16, 1.25) | <0.001 | 1.19 (1.12, 1.26) | <0.001 | 1.22 (1.16, 1.28) | <0.001 | 0.982 |
| Incontinence | 1.14 (1.12, 1.17) | <0.001 | 1.13 (1.09, 1.17) | <0.001 | 1.15 (1.11, 1.18) | <0.001 | 0.453 |
| Decubital ulcers | 1.35 (1.31, 1.40) | <0.001 | 1.39 (1.32, 1.46) | <0.001 | 1.33 (1.27, 1.38) | <0.001 | 0.142 |
| Malnutrition and malnourishment | 1.39 (1.32, 1.46) | <0.001 | 1.47 (1.35, 1.60) | <0.001 | 1.34 (1.25, 1.43) | <0.001 | 0.108 |
| Fluids and electrolytes disorders | 1.22 (1.19, 1.25) | <0.001 | 1.19 (1.15, 1.24) | <0.001 | 1.24 (1.20, 1.28) | <0.001 | 0.174 |
| Depression and anxiety disorders | 1.07 (1.05, 1.09) | <0.001 | 1.08 (1.05, 1.12) | <0.001 | 1.06 (1.03, 1.09) | <0.001 | 0.349 |
| Pain | 0.92 (0.90, 0.94) | <0.001 | 0.9 (0.87, 0.93) | <0.001 | 0.95 (0.92, 0.97) | <0.001 | 0.015 |
| Sensory disorders | 0.99 (0.97, 1.01) | 0.315 | 1.02 (0.98, 1.05) | 0.382 | 0.97 (0.94, 1.00) | 0.034 | 0.04 |
| Frailty | 1.00 (0.97, 1.03) | 0.92 | 0.99 (0.94, 1.04) | 0.615 | 1.01 (0.97, 1.05) | 0.621 | 0.488 |
| Severe visual and hearing impairment | 0.85 (0.84, 0.87) | <0.001 | 0.85 (0.82, 0.87) | <0.001 | 0.86 (0.84, 0.89) | <0.001 | 0.428 |
| Medication problems | 0.97 (0.88, 1.07) | 0.554 | 0.94 (0.81, 1.09) | 0.415 | 1.00 (0.88, 1.14) | 0.987 | 0.615 |
| High risk of complications | 1.02 (1.00, 1.05) | 0.072 | 0.98 (0.94, 1.01) | 0.214 | 1.06 (1.03, 1.10) | <0.001 | 0.006 |
| Delayed convalescence | 0.87 (0.76, 1.01) | 0.065 | 0.85 (0.68, 1.05) | 0.126 | 0.89 (0.74, 1.08) | 0.243 | 0.75 |
| Age in years at PHF diagnosis | 1.08 (1.08, 1.08) | <0.001 | 1.08 (1.08, 1.08) | <0.001 | 1.08 (1.08, 1.08) | <0.001 |  |
| Female Sex | 0.65 (0.63, 0.67) | <0.001 | 0.64 (0.62, 0.67) | <0.001 | 0.66 (0.64, 0.68) | <0.001 |  |
| Cancer | 1.06 (1.04, 1.08) | <0.001 | 1.08 (1.05, 1.12) | <0.001 | 1.04 (1.01, 1.07) | 0.004 |  |
| Diabetes | 1.22 (1.19, 1.24) | <0.001 | 1.22 (1.18, 1.26) | <0.001 | 1.21 (1.18, 1.25) | <0.001 |  |
| Dementia | 1.42 (1.37, 1.48) | <0.001 | 1.47 (1.39, 1.56) | <0.001 | 1.38 (1.32, 1.45) | <0.001 |  |
| Chronic polyarthritis | 1.08 (1.04, 1.13) | <0.001 | 1.11 (1.04, 1.18) | 0.001 | 1.06 (1.01, 1.12) | 0.03 |  |
| Obesity | 0.94 (0.92, 0.97) | <0.001 | 0.93 (0.90, 0.97) | <0.001 | 0.95 (0.92, 0.99) | 0.007 |  |
| Nicotine abuse | 1.43 (1.37, 1.49) | <0.001 | 1.41 (1.32, 1.50) | <0.001 | 1.45 (1.37, 1.54) | <0.001 |  |
| Parkinson | 1.31 (1.26, 1.37) | <0.001 | 1.26 (1.18, 1.35) | <0.001 | 1.34 (1.27, 1.42) | <0.001 |  |
| Rotator cuff rupture | 0.89 (0.83, 0.95) | <0.001 | 0.97 (0.89, 1.05) | 0.434 | 0.80 (0.72, 0.90) | <0.001 |  |
| Alcohol abuse | 1.52 (1.46, 1.59) | <0.001 | 1.54 (1.44, 1.65) | <0.001 | 1.51 (1.42, 1.60) | <0.001 |  |
| Previous stroke | 1.09 (1.06, 1.11) | <0.001 | 1.05 (1.02, 1.09) | 0.005 | 1.11 (1.07, 1.14) | <0.001 |  |
| Omarthrosis | 0.96 (0.9, 1.02) | 0.204 | 0.99 (0.89, 1.10) | 0.857 | 0.94 (0.87, 1.02) | 0.124 |  |
| Frozen shoulder | 0.86 (0.81, 0.9) | <0.001 | 0.87 (0.8, 0.95) | 0.001 | 0.85 (0.79, 0.90) | <0.001 |  |
| Atrial fibrillation and atrial flutter | 1.16 (1.13, 1.19) | <0.001 | 1.21 (1.16, 1.26) | <0.001 | 1.12 (1.08, 1.16) | <0.001 |  |
| Congestive heart failure | 1.19 (1.16, 1.22) | <0.001 | 1.18 (1.13, 1.22) | <0.001 | 1.20 (1.16, 1.23) | <0.001 |  |
| Coronary heart disease | 1.00 (0.98, 1.03) | 0.711 | 1.00 (0.96, 1.03) | 0.897 | 1.01 (0.98, 1.04) | 0.609 |  |
| Hypertonus | 1.05 (1.02, 1.08) | 0.002 | 1.08 (1.02, 1.13) | 0.003 | 1.03 (0.99, 1.08) | 0.114 |  |
| Atherosclerosis | 1.02 (0.99, 1.05) | 0.186 | 1.03 (0.99, 1.07) | 0.192 | 1.01 (0.98, 1.05) | 0.498 |  |
| Chronic kidney disease | 1.17 (1.15, 1.20) | <0.001 | 1.20 (1.15, 1.24) | <0.001 | 1.16 (1.12, 1.19) | <0.001 |  |
| Any anticoagulant | 1.12 (1.09, 1.14) | <0.001 | 1.10 (1.06, 1.14) | <0.001 | 1.12 (1.09, 1.16) | <0.001 |  |
| Vitamin D or Calcium | 0.99 (0.90, 1.08) | 0.806 | 0.99 (0.86, 1.13) | 0.856 | 0.99 (0.88, 1.11) | 0.836 |  |
| Bisphosphonate | 0.96 (0.87, 1.05) | 0.318 | 1.00 (0.87, 1.15) | 0.977 | 0.93 (0.82, 1.04) | 0.208 |  |
| Osteoporosis | 0.98 (0.96, 1.01) | 0.167 | 0.98 (0.94, 1.02) | 0.259 | 0.99 (0.96, 1.02) | 0.4 |  |
| Any osteoporosis medication | 1.15 (1.05, 1.28) | 0.004 | 1.15 (0.98, 1.34) | 0.082 | 1.16 (1.02, 1.32) | 0.024 |  |
| Outpatient sector | 0.87 (0.85, 0.89) | <0.001 | 1.34 (1.00, 1.79) | 0.046 | 0.81 (0.79, 0.83) | <0.001 |  |
| Treatment change within 21 days after diagnosis | 0.97 (0.90, 1.03) | 0.313 | 1.01 (0.94, 1.08) | 0.8 |  |  |  |
| Thromboembolic event within 21 days after diagnosis | 1.12 (0.66, 1.89) | 0.68 | 1.55 (0.57, 4.23) | 0.392 | 0.96 (0.52, 1.76) | 0.885 |  |
| Injury-related event within 21 days after diagnosis | 0.84 (0.78, 0.91) | <0.001 | 0.86 (0.75, 0.98) | 0.023 | 0.80 (0.73, 0.88) | <0.001 |  |
| Major adverse event within 21 days after diagnosis | 1,62 (1,21, 2,17) | 0,001 | 1,63 (0,86, 3,07) | 0,132 | 1,62 (1.17, 2.25) | 0,004 |  |
| Minor outpatient event within 21 days after diagnosis | 0,87 (0,81, 0,92) | <0,001 | 0,86 (0.74, 1.00) | 0,051 | 0,89 (0.83, 0.96) | 0,001 |  |

Table S11: Results of three Cox regression models with operative complications (SC) as an outcome for the overall cohort. The subgroup of those who underwent surgery within 21 days of PHF diagnosis, and those who did not, including only patients who remained event-free after 3 months. Included variables are current GTMK at the time of PHF diagnosis, patient (risk) profile, and events that occurred within 21 days of PHF diagnosis with an end of follow-up 3 months after the start of observation. The difference between operative and non-operative treatment within individual GTMK is quantified with the interaction p value p_int. This comes from a Cox regression, which in addition to the influencing factors listed here, also includes the interaction between operation and the individual GTMK.

| **Operative Complications (SC)** | | | | | | | |
| --- | --- | --- | --- | --- | --- | --- | --- |
|  | **Total cohort** | | **Subgroup:  Operative Treatment** | | **Subgroup:  Non-Operative Treatment** | | **OP vs no OP** |
| **Variable** | **HR (95%-CI)** | **p** | **HR (95%-CI)** | **P** | **HR (95%-CI)** | **p** | **P_interaction_** |
| Immobility | 0.86 (0.70, 1.07) | 0.175 | 1.08 (0.83, 1.41) | 0.562 | 0.62 (0.44, 0.88) | 0.007 | 0.008 |
| Falling tendency | 1.05 (0.98, 1.12) | 0.191 | 0.98 (0.90, 1.08) | 0.72 | 1.15 (1.04, 1.27) | 0.008 | 0.017 |
| Cognitive deficits | 0.92 (0.80, 1.07) | 0.282 | 0.84 (0.68, 1.02) | 0.081 | 1.05 (0.85, 1.30) | 0.659 | 0.569 |
| Incontinence | 0.95 (0.88, 1.02) | 0.168 | 0.94 (0.85, 1.04) | 0.257 | 0.95 (0.84, 1.06) | 0.334 | 0.921 |
| Decubital ulcers | 0.97 (0.86, 1.11) | 0.692 | 1.01 (0.85, 1.19) | 0.952 | 0.95 (0.79, 1.14) | 0.575 | 0.475 |
| Malnutrition and malnourishment | 0.81 (0.63, 1.05) | 0.114 | 0.72 (0.50, 1.03) | 0.072 | 0.94 (0.65, 1.34) | 0.72 | 0.372 |
| Fluids and electrolytes disorders | 0.97 (0.90, 1.05) | 0.515 | 0.94 (0.85, 1.04) | 0.258 | 1.03 (0.92, 1.16) | 0.608 | 0.385 |
| Depression and anxiety disorders | 1.04 (0.98, 1.11) | 0.203 | 1.05 (0.96, 1.14) | 0.274 | 1.03 (0.94, 1.13) | 0.549 | 0.968 |
| Pain | 1.09 (1.02, 1.16) | 0.01 | 1.16 (1.06, 1.26) | <0.001 | 0.99 (0.90, 1.09) | 0.868 | 0.066 |
| Sensory disorders | 1.19 (1.12, 1.27) | <0.001 | 1.26 (1.15, 1.37) | <0.001 | 1.12 (1.01, 1.24) | 0.027 | 0.117 |
| Frailty | 0.96 (0.86, 1.07) | 0.507 | 0.94 (0.81, 1.09) | 0.381 | 1.01 (0.86, 1.19) | 0.868 | 0.793 |
| Severe visual and hearing impairment | 1.05 (0.99, 1.12) | 0.096 | 1.10 (1.01, 1.19) | 0.021 | 0.99 (0.90, 1.08) | 0.779 | 0.282 |
| Medication problems | 1.23 (0.89, 1.70) | 0.21 | 1.33 (0.85, 2.1) | 0.213 | 1.20 (0.75, 1.92) | 0.44 | 0.807 |
| High risk of complications | 1.16 (1.09, 1.25) | <0.001 | 1.12 (1.03, 1.23) | 0.012 | 1.19 (1.08, 1.32) | <0.001 | 0.562 |
| Delayed convalescence | 0.73 (0.39, 1.35) | 0.314 | 1.04 (0.52, 2.09) | 0.914 | 0.34 (0.08, 1.35) | 0.124 | 0.14 |
| Age in years at PHF diagnosis | 0.96 (0.96, 0.97) | <0.001 | 0.96 (0.95, 0.96) | <0.001 | 0.97 (0.96, 0.98) | <0.001 |  |
| Female Sex | 0.99 (0.91, 1.08) | 0.805 | 0.98 (0.87, 1.11) | 0.793 | 0.98 (0.86, 1.11) | 0.755 |  |
| Cancer | 1.03 (0.96, 1.10) | 0.398 | 1.02 (0.93, 1.11) | 0.682 | 1.04 (0.95, 1.15) | 0.391 |  |
| Diabetes | 1.00 (0.93, 1.07) | 0.965 | 0.96 (0.88, 1.05) | 0.376 | 1.05 (0.95, 1.17) | 0.335 |  |
| Dementia | 0.74 (0.63, 0.87) | <0.001 | 0.83 (0.67, 1.03) | 0.096 | 0.65 (0.51, 0.82) | <0.001 |  |
| Chronic polyarthritis | 1.11 (0.99, 1.23) | 0.063 | 1.05 (0.91, 1.22) | 0.489 | 1.18 (1.01, 1.38) | 0.042 |  |
| Obesity | 1.18 (1.10, 1.26) | <0.001 | 1.16 (1.05, 1.27) | 0.002 | 1.19 (1.07, 1.32) | 0.002 |  |
| Nicotine abuse | 0.97 (0.87, 1.09) | 0.643 | 0.99 (0.86, 1.14) | 0.893 | 0.93 (0.77, 1.12) | 0.44 |  |
| Parkinson | 1.12 (0.95, 1.32) | 0.173 | 0.92 (0.72, 1.16) | 0.477 | 1.39 (1.11, 1.75) | 0.004 |  |
| Rotator cuff rupture | 1.19 (1.02, 1.40) | 0.03 | 1.19 (0.97, 1.45) | 0.096 | 1.20 (0.92, 1.56) | 0.184 |  |
| Alcohol abuse | 1.19 (1.06, 1.34) | 0.004 | 0.98 (0.84, 1.15) | 0.833 | 1.59 (1.33, 1.91) | <0.001 |  |
| Previous stroke | 0.97 (0.9, 1.04) | 0.36 | 0.99 (0.89, 1.09) | 0.769 | 0.94 (0.85, 1.05) | 0.295 |  |
| Omarthrosis | 1.13 (0.96, 1.32) | 0.135 | 0.99 (0.78, 1.25) | 0.916 | 1.29 (1.04, 1.60) | 0.018 |  |
| Frozen shoulder | 1.20 (1.06, 1.36) | 0.003 | 1.08 (0.90, 1.29) | 0.425 | 1.32 (1.12, 1.57) | 0.001 |  |
| Atrial fibrillation and atrial flutter | 0.99 (0.90, 1.08) | 0.759 | 1.10 (0.97, 1.26) | 0.127 | 0.87 (0.75, 1.00) | 0.057 |  |
| Congestive heart failure | 0.96 (0.88, 1.04) | 0.312 | 0.98 (0.88, 1.10) | 0.749 | 0.92 (0.81, 1.04) | 0.177 |  |
| Coronary heart disease | 1.03 (0.95, 1.11) | 0.497 | 1.00 (0.90, 1.10) | 0.942 | 1.05 (0.94, 1.17) | 0.421 |  |
| Hypertonus | 1.03 (0.95, 1.11) | 0.528 | 0.98 (0.88, 1.09) | 0.729 | 1.07 (0.95, 1.21) | 0.234 |  |
| Atherosclerosis | 1.04 (0.96, 1.13) | 0.319 | 1.12 (1.01, 1.25) | 0.038 | 0.94 (0.83, 1.07) | 0.379 |  |
| Chronic kidney disease | 0.95 (0.88, 1.03) | 0.246 | 1.00 (0.90, 1.11) | 0.955 | 0.91 (0.80, 1.03) | 0.125 |  |
| Any anticoagulant | 1.01 (0.93, 1.09) | 0.893 | 0.99 (0.89, 1.11) | 0.914 | 1.03 (0.92, 1.16) | 0.584 |  |
| Vitamin D or Calcium | 1.00 (0.78, 1.29) | 0.973 | 1.18 (0.85, 1.66) | 0.325 | 0.87 (0.61, 1.26) | 0.463 |  |
| Bisphosphonate | 1.01 (0.79, 1.30) | 0.937 | 0.95 (0.68, 1.33) | 0.773 | 1.09 (0.75, 1.60) | 0.643 |  |
| Osteoporosis | 1.11 (1.03, 1.18) | 0.004 | 1.01 (0.93, 1.11) | 0.778 | 1.24 (1.12, 1.38) | <0.001 |  |
| Any osteoporosis medication | 1.09 (0.83, 1.44) | 0.542 | 1.00 (0.68, 1.45) | 0.98 | 1.16 (0.77, 1.77) | 0.473 |  |
| Outpatient sector | 0.80 (0.75, 0.86) | <0.001 | 1.28 (0.61, 2.68) | 0.521 | 0.85 (0.78, 0.94) | <0.001 |  |
| Treatment change within 21 days after diagnosis | 22.88 (21.09, 24.82) | <0.001 | 21.04 (19.3, 22.94) | <0.001 |  |  |  |
| Thromboembolic event within 21 days after diagnosis | 1.89 (0.15, 23.93) | 0.623 | 0.00 (0.00, Inf) | 0.991 | 1.74 (0.12, 25.88) | 0.687 |  |
| Injury-related event within 21 days after diagnosis | 1.42 (1.20, 1.68) | <0.001 | 1.39 (1.08, 1.79) | 0.01 | 1.47 (1.17, 1.84) | <0.001 |  |
| Major adverse event within 21 days after diagnosis | 0.52 (0.09, 3.11) | 0.471 | 0.00 (0.00, Inf) | 0.98 | 0.78 (0.12, 5.24) | 0.795 |  |
| Minor outpatient event within 21 days after diagnosis | 1.15 (1.00, 1.34) | 0.056 | 1.06 (0.79, 1.43) | 0.679 | 1.19 (0.99, 1.43) | 0.06 |  |

Table S12: Results of three Cox regression models with minor outpatient complications (MOC) as an outcome for the overall cohort. The subgroup of those who underwent surgery within 21 days of PHF diagnosis, and those who did not, including only patients who remained event-free after 3 months. Included variables are current GTMK at the time of PHF diagnosis, patient (risk) profile, and events that occurred within 21 days of PHF diagnosis with an end of follow-up 3 months after the start of observation. The difference between operative and non-operative treatment within individual GTMK is quantified with the interaction p value p_int. This comes from a Cox regression which, in addition to the influencing factors listed here, also includes the interaction between operation and the individual GTMK.

| **Minor Outpatient Complications (MOC)** | | | | | | | |
| --- | --- | --- | --- | --- | --- | --- | --- |
|  | **Total cohort** | | **Subgroup:  Operative Treatment** | | **Subgroup:  Non-Operative Treatment** | | **OP vs no OP** |
| **Variable** | **HR (95%-CI)** | **p** | **HR (95%-CI)** | **P** | **HR (95%-CI)** | **p** | **P_interaction_** |
| Immobility | 0.93 (0.84, 1.04) | 0.197 | 0.94 (0.81, 1.09) | 0.405 | 0.94 (0.81, 1.08) | 0.362 | 0.912 |
| Falling tendency | 1.07 (1.03, 1.11) | <0.001 | 1.04 (0.99, 1.09) | 0.142 | 1.11 (1.06, 1.17) | <0.001 | 0.14 |
| Cognitive deficits | 0.81 (0.75, 0.88) | <0.001 | 0.85 (0.76, 0.95) | 0.004 | 0.78 (0.70, 0.87) | <0.001 | 0.283 |
| Incontinence | 1.02 (0.98, 1.06) | 0.417 | 1.04 (0.98, 1.10) | 0.154 | 1.00 (0.95, 1.06) | 0.865 | 0.439 |
| Decubital ulcers | 0.91 (0.85, 0.98) | 0.009 | 0.95 (0.86, 1.04) | 0.262 | 0.90 (0.82, 0.99) | 0.032 | 0.536 |
| Malnutrition and malnourishment | 0.70 (0.60, 0.80) | <0.001 | 0.79 (0.65, 0.95) | 0.015 | 0.62 (0.50, 0.76) | <0.001 | 0.087 |
| Fluids and electrolytes disorders | 0.92 (0.89, 0.96) | <0.001 | 0.96 (0.9, 1.02) | 0.147 | 0.9 (0.85, 0.96) | <0.001 | 0.207 |
| Depression and anxiety disorders | 1.09 (1.06, 1.13) | <0.001 | 1.11 (1.06, 1.17) | <0.001 | 1.08 (1.03, 1.13) | 0.002 | 0.64 |
| Pain | 1.31 (1.27, 1.35) | <0.001 | 1.26 (1.21, 1.33) | <0.001 | 1.35 (1.29, 1.42) | <0.001 | 0.016 |
| Sensory disorders | 1.26 (1.22, 1.31) | <0.001 | 1.23 (1.17, 1.29) | <0.001 | 1.29 (1.23, 1.36) | <0.001 | 0.043 |
| Frailty | 0.94 (0.89, 1.00) | 0.048 | 0.97 (0.89, 1.05) | 0.435 | 0.93 (0.86, 1.00) | 0.055 | 0.187 |
| Severe visual and hearing impairment | 1.24 (1.20, 1.28) | <0.001 | 1.24 (1.18, 1.30) | <0.001 | 1.23 (1.18, 1.29) | <0.001 | 0.974 |
| Medication problems | 0.89 (0.73, 1.09) | 0.251 | 0.82 (0.62, 1.10) | 0.191 | 0.96 (0.73, 1.26) | 0.778 | 0.382 |
| High risk of complications | 1.15 (1.11, 1.19) | <0.001 | 1.17 (1.11, 1.23) | <0.001 | 1.13 (1.07, 1.18) | <0.001 | 0.397 |
| Delayed convalescence | 1.05 (0.81, 1.37) | 0.713 | 1.08 (0.75, 1.56) | 0.687 | 1.03 (0.7, 1.51) | 0.897 | 0.888 |
| Age in years at PHF diagnosis | 0.97 (0.97, 0.97) | <0.001 | 0.98 (0.97, 0.98) | <0.001 | 0.97 (0.97, 0.97) | <0.001 |  |
| Female Sex | 1.10 (1.05, 1.15) | <0.001 | 1.09 (1.01, 1.17) | 0.023 | 1.10 (1.03, 1.18) | 0.004 |  |
| Cancer | 1.06 (1.03, 1.10) | <0.001 | 1.00 (0.95, 1.05) | 0.97 | 1.12 (1.07, 1.18) | <0.001 |  |
| Diabetes | 0.92 (0.89, 0.96) | <0.001 | 0.91 (0.86, 0.96) | <0.001 | 0.93 (0.89, 0.98) | 0.009 |  |
| Dementia | 0.71 (0.65, 0.78) | <0.001 | 0.71 (0.63, 0.80) | <0.001 | 0.74 (0.66, 0.84) | <0.001 |  |
| Chronic polyarthritis | 1.09 (1.03, 1.15) | 0.005 | 1.09 (1.00, 1.19) | 0.048 | 1.09 (1.01, 1.19) | 0.031 |  |
| Obesity | 1.14 (1.09, 1.18) | <0.001 | 1.12 (1.06, 1.18) | <0.001 | 1.15 (1.09, 1.22) | <0.001 |  |
| Nicotine abuse | 0.98 (0.92, 1.04) | 0.518 | 1.00 (0.92, 1.10) | 0.922 | 0.95 (0.87, 1.04) | 0.283 |  |
| Parkinson | 0.91 (0.83, 1.00) | 0.041 | 0.79 (0.68, 0.91) | <0.001 | 1.04 (0.92, 1.18) | 0.56 |  |
| Rotator cuff rupture | 1.23 (1.12, 1.36) | <0.001 | 1.19 (1.05, 1.35) | 0.007 | 1.21 (1.03, 1.40) | 0.017 |  |
| Alcohol abuse | 0.97 (0.90, 1.05) | 0.441 | 0.97 (0.88, 1.08) | 0.618 | 0.96 (0.86, 1.08) | 0.525 |  |
| Previous stroke | 1.00 (0.96, 1.04) | 0.874 | 1.01 (0.96, 1.07) | 0.664 | 1.00 (0.95, 1.06) | 0.959 |  |
| Omarthrosis | 1.56 (1.44, 1.69) | <0.001 | 1.44 (1.26, 1.64) | <0.001 | 1.65 (1.49, 1.83) | <0.001 |  |
| Frozen shoulder | 1.80 (1.68, 1.92) | <0.001 | 1.65 (1.49, 1.83) | <0.001 | 1.93 (1.77, 2.11) | <0.001 |  |
| Atrial fibrillation and atrial flutter | 0.91 (0.86, 0.95) | <0.001 | 0.92 (0.86, 0.99) | 0.028 | 0.9 (0.84, 0.97) | 0.004 |  |
| Congestive heart failure | 1.01 (0.97, 1.05) | 0.688 | 0.99 (0.93, 1.06) | 0.833 | 1.03 (0.97, 1.1) | 0.28 |  |
| Coronary heart disease | 1.08 (1.04, 1.12) | <0.001 | 1.07 (1.01, 1.13) | 0.023 | 1.09 (1.03, 1.15) | 0.004 |  |
| Hypertonus | 1.04 (1.00, 1.09) | 0.077 | 1.04 (0.98, 1.11) | 0.2 | 1.03 (0.97, 1.10) | 0.284 |  |
| Atherosclerosis | 1.05 (1.00, 1.09) | 0.038 | 1.05 (0.99, 1.12) | 0.117 | 1.04 (0.98, 1.11) | 0.182 |  |
| Chronic kidney disease | 1.02 (0.98, 1.07) | 0.312 | 1.02 (0.96, 1.09) | 0.438 | 1.02 (0.96, 1.08) | 0.506 |  |
| Any anticoagulant | 0.95 (0.91, 0.99) | 0.021 | 0.93 (0.87, 0.99) | 0.017 | 0.99 (0.93, 1.04) | 0.616 |  |
| Vitamin D or Calcium | 1.02 (0.89, 1.17) | 0.751 | 1.06 (0.87, 1.28) | 0.562 | 0.99 (0.82, 1.20) | 0.919 |  |
| Bisphosphonate | 0.98 (0.86, 1.13) | 0.794 | 0.95 (0.79, 1.16) | 0.641 | 1.00 (0.83, 1.22) | 0.984 |  |
| Osteoporosis | 1.11 (1.07, 1.15) | <0.001 | 1.11 (1.05, 1.17) | <0.001 | 1.10 (1.04, 1.16) | <0.001 |  |
| Any osteoporosis medication | 1.04 (0.90, 1.22) | 0.579 | 1.01 (0.81, 1.26) | 0.915 | 1.08 (0.87, 1.34) | 0.473 |  |
| Outpatient sector | 0.90 (0.87, 0.93) | <0.001 | 0.77 (0.48, 1.23) | 0.271 | 1.06 (1.01, 1.11) | 0.015 |  |
| Treatment change within 21 days after diagnosis | 2.25 (2.08, 2.43) | <0.001 | 2.09 (1.94, 2.27) | <0.001 |  |  |  |
| Thromboembolic event within 21 days after diagnosis | 0.63 (0.13, 2.93) | 0.552 | 0.00 (0.00, Inf) | 0.959 | 0.78 (0.15, 3.98) | 0.761 |  |
| Injury-related event within 21 days after diagnosis | 1.42 (1.28, 1.58) | <0.001 | 1.34 (1.14, 1.59) | <0.001 | 1.65 (1.43, 1.89) | <0.001 |  |
| Major adverse event within 21 days after diagnosis | 0.72 (0.33, 1.56) | 0.403 | 0.75 (0.19, 2.99) | 0.68 | 0.77 (0.3, 1.98) | 0.585 |  |
| Minor outpatient event within 21 days after diagnosis | 1.63 (1.51, 1.77) | <0.001 | 1.4 (1.16, 1.69) | <0.001 | 1.69 (1.54, 1.85) | <0.001 |  |

**Literature:**

1. Katthagen JC. Raschke MJ. Fischhuber K. et al. Conservative Versus Operative Treatment of Proximal Humerus Fractures in Older Individuals—an Analysis of Insurance Data. *Dtsch Arzteblatt Int*. 2024,(Forthcoming):arztebl.m2024.0059. doi:10.3238/arztebl.m2024.0059

2. Koeppe J. Stolberg-Stolberg J. Rischen R. et al. Increased complication rates of salvage reverse total shoulder arthroplasty (RTSA) after failed locked plate fixation compared with primary RTSA in the treatment of proximal humeral fractures in elderly patients. *J Shoulder Elbow Surg*. 2023,32(8):1574-1583. doi:10.1016/j.jse.2022.12.020

3. Abgrenzungskriterien der Geriatrie - Version V1.3.

1. These codes were only collected if they were coded within 3 months prior to the index event. [↑](#footnote-ref-1)
2. Fractures (codes of the form S-2.-[-]) were only recorded if they were coded within 26 weeks prior to the index event. [↑](#footnote-ref-2)
3. Dialysis requirement is assumed if at least 12 dialyses were coded within 2 years prior to the index event. [↑](#footnote-ref-3)
